# Restricted glycolysis is a primary cause of the reduced growth rate of zinc-deficient yeast cells

**DOI:** 10.1016/j.jbc.2024.107147

**Published:** 2024-03-07

**Authors:** Colin W. MacDiarmid, Janet Taggart, Michael Kubisiak, David J. Eide

**Affiliations:** Department of Nutritional Sciences, University of Wisconsin-Madison, Madison, Wisconsin, USA

**Keywords:** zinc, *Saccharomyces cerevisiae*, glycolysis, pyruvate kinase, peroxiredoxin

## Abstract

Zinc is required for many critical processes, including intermediary metabolism. In *Saccharomyces cerevisiae*, the Zap1 activator regulates the transcription of ∼80 genes in response to Zn supply. Some Zap1-regulated genes are Zn transporters that maintain Zn homeostasis, while others mediate adaptive responses that enhance fitness. One adaptive response gene encodes the 2-cysteine peroxiredoxin Tsa1, which is critical to Zn-deficient (ZnD) growth. Depending on its redox state, Tsa1 can function as a peroxidase, a protein chaperone, or a regulatory redox sensor. In a screen for possible Tsa1 regulatory targets, we identified a mutation (*cdc19*^*S492A*^) that partially suppressed the *tsa1Δ* growth defect. The *cdc19*^*S492A*^ mutation reduced activity of its protein product, pyruvate kinase isozyme 1 (Pyk1), implicating Tsa1 in adapting glycolysis to ZnD conditions. Glycolysis requires activity of the Zn-dependent enzyme fructose-bisphosphate aldolase 1, which was substantially decreased in ZnD cells. We hypothesized that in ZnD *tsa1Δ* cells, the loss of a compensatory Tsa1 regulatory function causes depletion of glycolytic intermediates and restricts dependent amino acid synthesis pathways, and that the decreased activity of Pyk1^S492A^ counteracted this depletion by slowing the irreversible conversion of phosphoenolpyruvate to pyruvate. In support of this model, supplementing ZnD *tsa1Δ* cells with aromatic amino acids improved their growth. Phosphoenolpyruvate supplementation, in contrast, had a much greater effect on growth rate of WT and *tsa1Δ* ZnD cells, indicating that inefficient glycolysis is a major factor limiting yeast growth. Surprisingly however, this restriction was not primarily due to low fructose-bisphosphate aldolase 1 activity, but instead occurs earlier in glycolysis.

Zinc (Zn) is an essential nutrient for all organisms (1, 2). As a structural and/or catalytic cofactor, it is required for the function of a significant fraction of proteins (3, 4, 5). Examination of genomic sequences has indicated that ∼5% of genes in prokaryotes and 10% of eukaryotic genes encode Zn-dependent proteins (6). Many Zn-binding proteins are essential for cell growth and viability, including transcription factor IIIA (7), RNA polymerase (8), ribosome subunits (9), and many other enzymes (10). The importance of Zn to cell viability is readily apparent when comparing growth of Zn-replete (ZnR) *versus* Zn-deficient (ZnD) cells; Zn deficiency greatly reduces growth rate (11). Despite the many critical roles of Zn, however, there is little information on how Zn limitation restricts fitness of eukaryotic cells and which processes are most directly responsible.

The importance of Zn for cell growth is exploited by the immune system to combat microbial infections (2). In response to bacterial or fungal infection, substantial amounts of Zn are removed from the bloodstream and stored in the liver in a process known as “nutritional immunity” (12, 13). By reducing the level of circulating Zn available for growth of pathogens, our bodies can better resist infection. Another mechanism for resisting microbial pathogens relies on the Zn-binding protein calprotectin (14), an abundant antimicrobial protein released by neutrophils and epithelial cells at sites of infection. Binding of Zn by calprotectin further limits the availability of this essential nutrient to the invading microbe. Thus, the regulation of Zn availability to microbes is a key component of innate immunity.

Among fungi, processes of Zn transport and trafficking and how they are regulated are arguably best understood in the yeast *Saccharomyces cerevisiae* (1, 15). A large part of the yeast transcriptional response to Zn deficiency is orchestrated by the Zap1 transcriptional activator. Zap1 regulates the expression of over 80 genes that fall into two broad categories, those mediating the homeostatic response (*e.g.* specific transporters responsible for Zn uptake into the cell and its trafficking between compartments) (1) and those for the adaptive response (which do not affect Zn availability, but help cells better tolerate the stress of Zn deficiency) (15, 16). Adaptive response factors include enzymes that help maintain phospholipid synthesis in low Zn (17, 18), antioxidant proteins that help ZnD cells maintain redox homeostasis (19), and factors affecting the stability of the Zn proteome (20, 21, 22).

One important Zap1-regulated adaptive response gene encodes the peroxiredoxin (PR) Tsa1 (20, 23). PRs are a ubiquitous family of multifunctional cysteine-dependent peroxidase enzymes with extremely high affinity for their substrates (hydrogen peroxide [H_2_O_2_], alkyl hydroperoxides, and peroxynitrite) (24, 25, 26, 27, 28). Like other "typical" 2-Cys PR, Tsa1 has several functions. First, as a peroxidase, the active site "peroxidatic" Cys thiol (C48, SH_P_) reacts with H_2_O_2_ or other substrates to form a sulfenate side chain. In turn this reacts with the "resolving" Cys thiol (C171, SH_R_) of a second bound monomer to generate a disulfide-linked homodimer, which can then be reduced by thioredoxin (29). Tsa1 thus participates in the maintenance of low intracellular reactive oxygen species (ROS) and reactive nitrogen species (RNS) driven by NADPH consumption. Second, at higher substrate concentrations SH_P_ can be hyperoxidized to a sulfenic or sulfonic acid, inactivating the peroxidase but activating a holdase-type protein chaperone function (30, 31). Third, Tsa1 has a "redox sensor" function (32). In this role, the SH_P_ cysteine is oxidized by substrate, and this oxidation is then transferred to cysteines of physically interacting target proteins *via* formation of an intermolecular disulfide crosslink. Reaction of this crosslink with a second target protein cysteine resolves the complex into reduced Tsa1 and oxidized target. While the redox relay function of PR enzymes has been known for some time (33, 34, 35, 36), few *bona fide* targets for PR regulators have been identified, and the full importance of PR redox sensor function remains unclear.

Tsa1 is essential for growth of *S. cerevisiae* in severely ZnD media. In an effort to better understand Tsa1's important role in adaptation, we conducted a genetic suppressor screen to identify essential Tsa1-dependent processes. As described in this report, this analysis led to the exciting discovery of a role for Tsa1 in the adaptation of glycolysis to low Zn. ZnD *tsa1Δ* mutants displayed an aromatic amino acid auxotrophy consistent with a restricted supply of the critical glycolytic intermediate phosphoenolpyruvate (PEP). Surprisingly however, supplementation with PEP strongly enhanced the growth of both *tsa1Δ* and WT ZnD cells, identifying inefficient glycolysis as the predominant factor restricting fitness under ZnD conditions.

## Results

**Figure 1:**
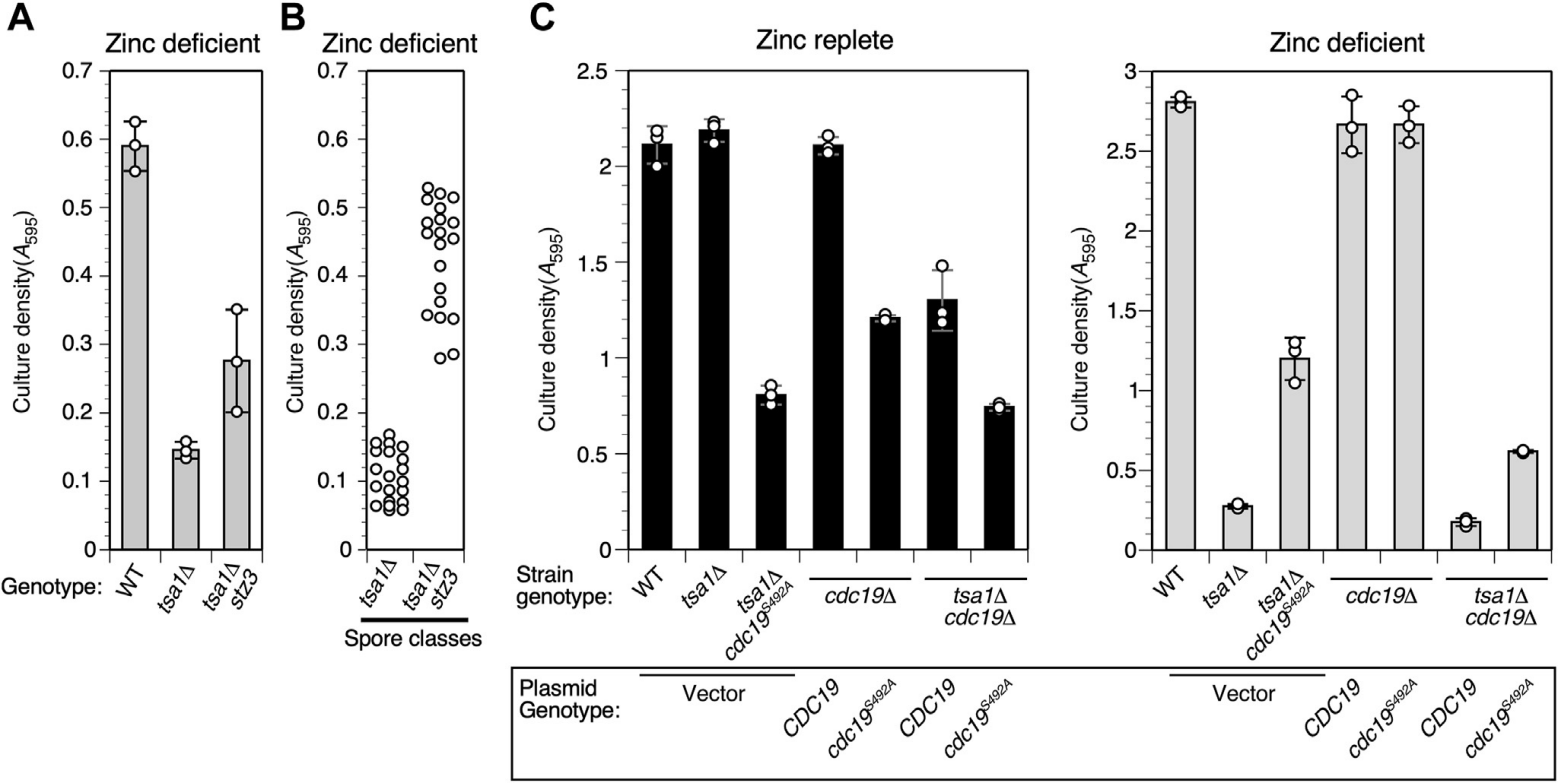
**Identification of the *cdc19***^***S492A***^**suppressor mutation.***A*, effect of Zn deficiency on growth of the *tsa1Δ stz3* suppressor mutant strain (clone 23-1D/22) compared to CWY2 (WT) and progenitor CWY8 (*tsa1Δ*). *B*, growth in low Zn of the 20 spore clones in each phenotypic pool (single *tsa1Δ versus* double *tsa1Δ stz3* genotype) as sorted for whole genome sequencing. Spore clones were derived from 10 tetrads obtained by sporulating a 23-1D/22 x CWY8 diploid. The full set of growth data is shown in Fig. S1. *C*, effect of Zn deficiency on growth of CWY2 (WT), CWY8 (*tsa1Δ*), 23-1D/22 (*tsa1Δ cdc19*^*S492A*^), CWM313 (*cdc19Δ* + *pCDC19*), CWM314 (*cdc19Δ* + *pCDC19*^*S492A*^), CWM327 (*cdc19Δ tsa1Δ* + *pCDC19*), and CWM329 (*cdc19Δ tsa1Δ* + *pCDC19*^*S492A*^) yeast strains. LZM cultures were inoculated to 0.01 starting *A*_595_ and incubated for 1 day in ZnR (*left* panel, LZM + 100 μM Zn) or 3 days in ZnD medium (*right* panel, LZM + 1 μM Zn). For *A* and *C*, columns show the average of three experimental replicates and error bars indicate ± 1 SD. For B, datapoints are the average of three experimental replicates. LZM, low Zn medium; ZnD, Zn-deficient; ZnR, Zn-replete.

To identify processes disrupted in ZnD *tsa1Δ* mutants that are responsible for their slow growth compared to WT cells, we performed a genetic screen for suppressor mutations that improved the growth of ZnD *tsa1Δ* cells (20). In this screen, we identified a gain-of-function mutation in the *TSA1* paralog *TSA2* that increased its expression, and a loss-of-function mutation in the *TRR1* thioredoxin reductase gene. We found that *TRR1* inactivation increased expression of the Yap1-regulated gene *TSA2*, and we proposed that the increased expression of *TSA2* compensated for the missing Tsa1 activity. The same genetic screen also identified a third complementation group (*stz3,* suppressor of *tsa1* zinc phenotype). As shown in Figure 1*A*, *stz3* partially restored the growth of a *tsa1Δ* mutant under ZnD conditions. To identify the *stz3* mutation, we performed a pooled linkage analysis combined with whole genome sequencing (WGS). Dissection of 10 tetrads derived from a *tsa1Δ*/*tsa1Δ stz3/+* diploid yielded 40 haploid segregants, which were sorted into suppressed (*i.e.*, *tsa1Δ stz3* genotype) and WT *(tsa1Δ STZ3* genotype) categories. The suppressor phenotype segregated 2:2 in these tetrads consistent with a mutation at a single locus (Figs. 1*B* and S1). Spore clones were combined into WT and *stz3* pools, and genomic DNA was isolated from each pool for WGS. This analysis identified a single nucleotide polymorphism on chromosome I (T73259G) that differed from the reference sequence and was found only in the *stz3* spore pool (Table S1). This SNP was located in *CDC19* (which encodes pyruvate kinase isozyme 1 [Pyk1]), and the mutation altered serine 492 to alanine (*cdc19*^*S492A*^). Throughout this article, we will refer to the affected gene as *CDC19* and its protein product as Pyk1.

To verify that *cdc19*^*S492A*^ was responsible for suppression, *cdc19Δ* and *cdc19Δ tsa1Δ* strains carrying plasmids encoding either WT *CDC19* or the *cdc19*^*S492A*^ allele were grown in ZnR and ZnD conditions. The *cdc19*^*S492A*^ strains grew more slowly than the WT transformants in ZnR medium (Fig. 1*C*, ZnR) suggesting that the mutation reduced gene function. Consistent with this observation, complementation experiments indicated that *cdc19*^*S492A*^ was recessive (Fig. S1*B*). In ZnD medium however, *tsa1Δ* cells expressing *cdc19*^*S492A*^ grew faster than those expressing WT *CDC19* (Fig. 1*C*), confirming that *cdc19*^*S492A*^ is responsible for the suppression phenotype.

**Figure 2:**
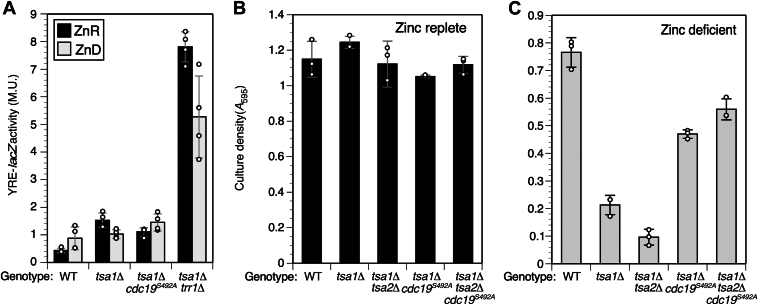
***cdc19***^***S492A***^**does not activate Yap1 and suppression is independent of Tsa2.***A*, activity of a Yap1-regulated lacZ reporter gene (pJAW79) was determined in strains of the indicated genotypes after growth in ZnR (100 μM Zn) or ZnD (1 μM Zn) LZM and harvest at log phase. Strains used were CWY2 (WT), CWY8 (*tsa1Δ*), 23-1D/7 (*tsa1Δ cdc19*^*S492A*^*)* and CWM83 (*tsa1Δ trr1Δ*). MU = Miller units. *B*, strains of the indicated genotypes were used to inoculate LZM medium to an initial *A*_595_ of 0.01. *A*_595_ was measured after 1 day in ZnR medium (*left* panel) or 2 days in deficient medium (*right* panel). Yeast strains used were CWY2 (WT), CWY8 (*tsa1Δ*), CWY10 (*tsa1Δ tsa2Δ*), 23-1d/22 (*tsa1Δ cdc19*^*S492A*^), and MDY2 (*tsa1Δ tsa2Δ cdc19*^*S492A*^). For panels *B* and *C*, columns are the average of three experimental replicates (for panel *A*, four replicates) and error bars indicate ± 1 SD. LZM, low Zn medium; ZnD, Zn-deficient; ZnR, Zn-replete.

Inactivation of *TRR1* suppressed *tsa1Δ* via elevated Yap1 activity and a resulting increase in *TSA2* expression. To determine if *cdc19*^*S492A*^ suppressed *tsa1Δ* via a related mechanism, we first tested its effect on Yap1 activity. As previously observed (20), a *tsa1Δ trr1Δ* mutant showed elevated activity of a Yap1-responsive *lacZ* reporter gene relative to *tsa1Δ* cells, in both ZnR and ZnD conditions (Fig. 2*A*). In contrast, the *cdc19*^*S492A*^ allele had only a small effect on Yap1 activity. We then tested for dependence of suppression on the *TSA2* gene by comparing growth of a *tsa1Δ cdc19*^*S492A*^ strain with a *tsa1Δ tsa2Δ cdc19*^*S492A*^ strain in ZnR and ZnD conditions (Fig. 2*BC*). This comparison indicated that in contrast to the *trr1Δ* suppressor mutation, *TSA2* was not required for suppression by *cdc19*^*S492A*^. Therefore, we concluded that the *cdc19*^*S492A*^ allele suppressed *tsa1Δ* via a novel mechanism.

**Figure 3:**
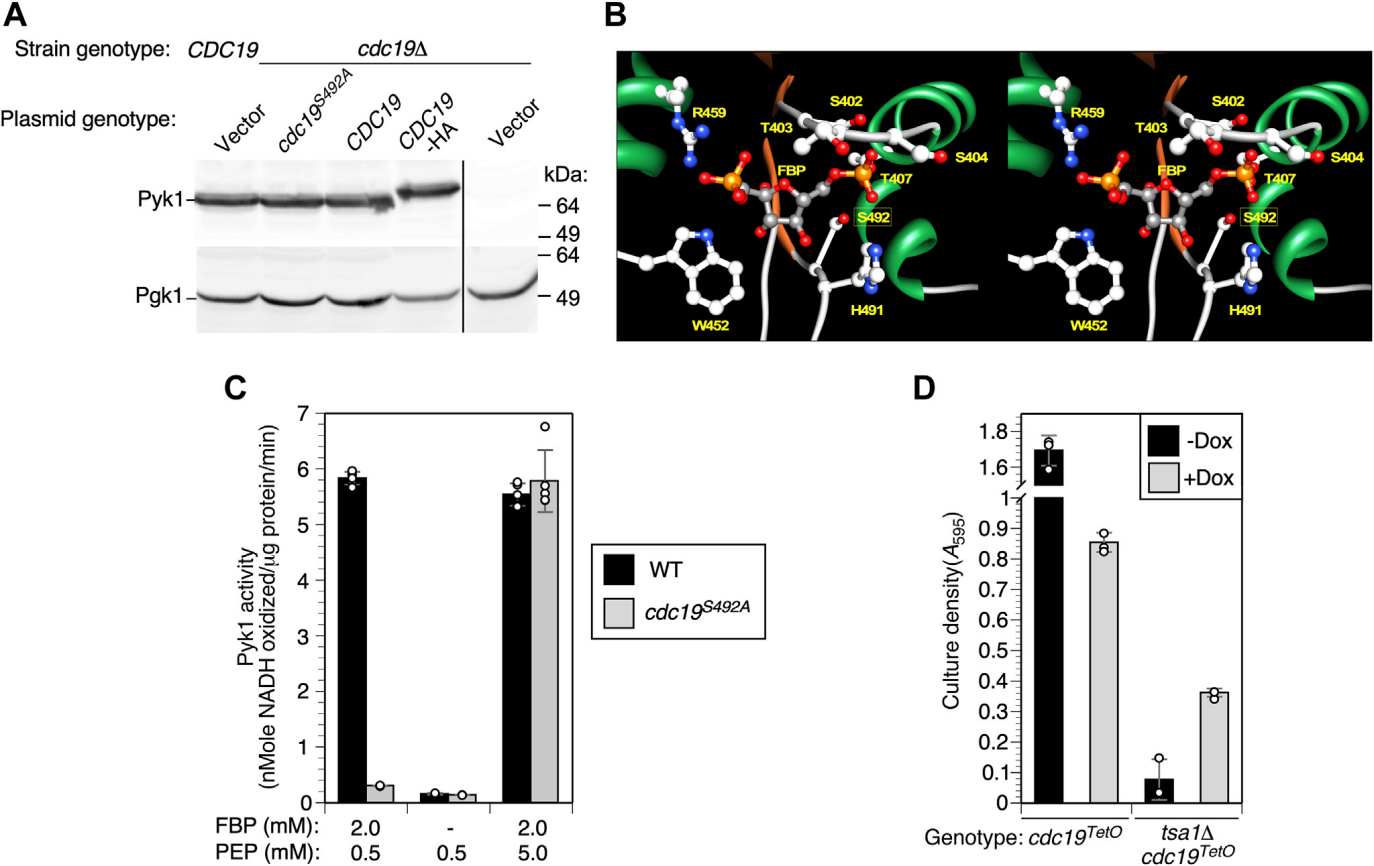
**Effect of the *cdc19***^***S492A***^**mutation on pyruvate kinase activity.***A*, the *cdc19*^*S492A*^ mutation does not affect Pyk1 accumulation. Total protein extracted from strains of the indicated genotypes was analyzed by SDS-PAGE and immunoblotting with an anti-Pyk1 antibody. Cells were grown in YPGE medium to enable growth of the vector only deletion mutant control. Strains were BY4742 and CWM307 (BY4742 *cdc19Δ*) transformed with pFL38 (empty vector), *pCDC19*, p*cdc19*^*S492A*^, or *pCDC19*-HA. *Black line* indicates deleted lane of molecular mass marker. *B*, Walleye stereo plot of the Pyk1 FBP allosteric site (PDB model 1A3W) (37) showing the location of the residues making contacts with FBP (*yellow labels*), and of S492 (*boxed*). *C*, pyruvate kinase (Pyk) activity was measured in extracts of ZnR cells of the genotypes indicated (strains CWM313 and 314). Assays were performed with the indicated substrate (PEP) and activator (FBP) concentrations. *D*, growth of strains carrying *cdc19*^*TetO_7_*^, an allele repressible by addition of doxycycline. *TSA1 cdc19*^*TetO_7_*^ (TH4015) or *tsa1Δ cdc19*^*TetO_7_*^ (CWM343) strains were grown to saturation in SD medium ± 1 μg/ml doxycycline, and then used to inoculate ZnD medium ± 2 μg/ml doxycycline to an initial A_595_ of 0.01. Cultures were grown for 5 days before measurement of cell density. Columns are the average of five (panel *C*) or three (panel *D*) experimental replicates and error bars indicate ± 1 SD. FBP, fructose 1,6-bisphosphate; PDB, Protein Data Bank; PEP, phosphoenolpyruvate; SD medium, synthetic defined medium; YPGE, yeast extract peptone medium plates with 3% glycerol and 2% ethanol; ZnD, Zn-deficientZnR, Zn-replete; .

To explore the mechanism of suppression mediated by the *cdc19*^*S492A*^ allele, we tested the effect of this mutation on the protein encoded by *CDC19*, Pyk1. Pyk1 converts PEP into pyruvate during glycolysis. The recessive nature of *cdc19*^*S492A*^ and its effect on fitness of ZnR cells suggested that it decreased Pyk1 activity. This effect was not due to decreased abundance as the *cdc19*^*S492A*^ mutation did not affect Pyk1 accumulation (Fig. 3*A*). A possible explanation for the effect of *cdc19*^*S492A*^ was suggested by the location of S492 near the binding site for fructose 1,6-bisphosphate (FBP), the major allosteric activator of Pyk1 (Fig. 3*B*) (37). The side chain of S492 projects into the FBP-binding pocket and is immediately adjacent to H491, a residue that makes a hydrogen bond with FBP. We suspected that replacing the polar S492 residue with a hydrophobic residue might impair FBP binding and disrupt allosteric activation. To test this prediction, we measured pyruvate kinase specific activity with or without FBP, using a moderate PEP concentration (0.5 mM) to reveal allosteric activation. In the absence of FBP, Pyk1 activity was low in both WT and *cdc19*^*S492A*^ cell extracts (Fig. 3*C*). Inclusion of 2 mM FBP greatly increased WT Pyk1 activity but had little effect on Pyk1^S492A^. Binding of FBP to Pyk1 activates the enzyme by increasing its affinity for PEP (38, 39). With 2 mM FBP and a higher PEP concentration (5 mM), a similar level of activity was measured for both WT and Pyk1^S492A^ enzymes. These results indicate that the *cdc19*^*S492A*^ mutation does not disrupt Pyk1 basal activity, but rather specifically prevents its allosteric activation by FBP.

It was conceivable that *cdc19*^*S492A*^ suppressed *tsa1Δ* via a mechanism unrelated to reduced Pyk1 activity. To investigate this possibility, we used a strain in which the *CDC19* promoter was replaced with the doxycycline-repressible TetO_7_ promoter (*cdc19*^*TetO_7_*^*)*, which enabled the repression of WT Pyk1 expression by addition of doxycycline (40). In ZnD *TSA1 cdc19*^*TetO_7_*^ cells, doxycycline treatment slowed growth considerably, consistent with restricted Pyk1 activity (Figs. 3*D* and S2*A*). In contrast, ZnD *tsa1Δ cdc19*^*TetO_7_*^ cells grew slowly in the absence of doxycycline, but repression of Pyk1 expression with doxycycline markedly improved growth. These data are consistent with the hypothesis that decreased Pyk1 activity is the primary cause of *tsa1Δ* suppression by *cdc19*^*S492A*^. Further support for this hypothesis came from experiments in which the Pyk1 isozyme was replaced with Pyk2, which is not allosterically activated by FBP (41) and consequently has a lower specific activity (42). As *PYK2* expression is normally tightly repressed in glucose medium, it was expressed from the constitutive *TEF1* promoter. In ZnD medium, Pyk2 expression enhanced the growth of a *cdc19Δ tsa1Δ* mutant, while expression of Pyk1 from the *TEF1* promoter did not (Fig. S2*B*). The *TEF1pr-PYK2* allele was almost as effective at suppression as the *cdc19*^*S492A*^ mutation. Together these observations indicate that suppression was not due to some unknown property of the *cdc19*^*S492A*^ allele but was instead a consequence of its low pyruvate kinase activity.

**Figure 4:**
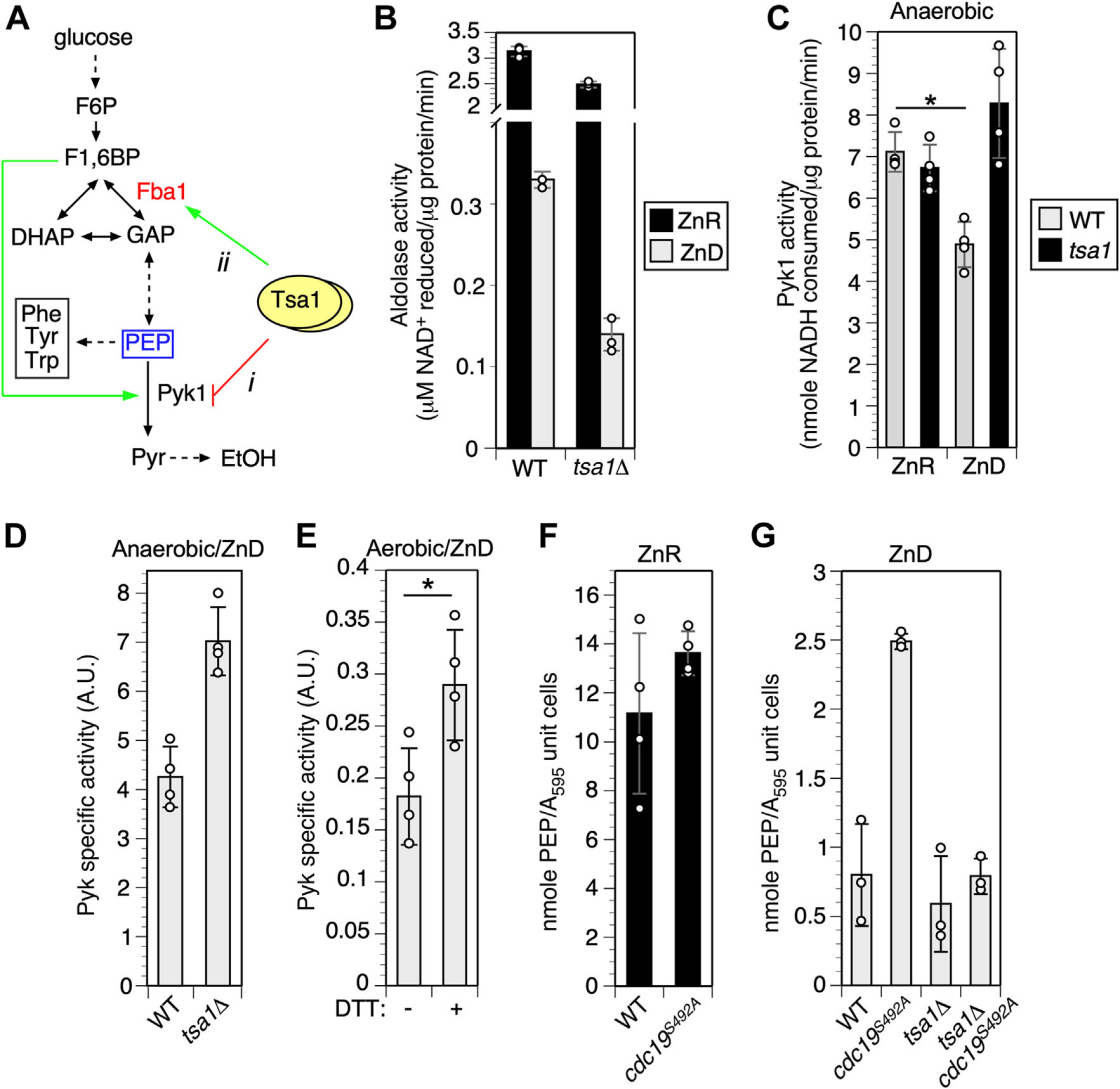
**Effect of zinc deficiency on Pyk1 activity and glycolytic intermediates.***A*, glycolytic pathway showing proposed regulatory roles of Tsa1 for Pyk1 (*i*, inhibition) and Fba1 (*ii*, activation) in ZnD cells. *B*, aldolase activity in ZnR and ZnD WT and *tsa1Δ* cells. WT (BY4742) and isogenic *tsa1Δ* strains were grown to log phase and aldolase activity measured in cell extracts. Assays were supplemented with 5 μM Zn. *C*, effect of low Zn and *tsa1Δ* mutation on Pyk1 activity. Protein extracts of ZnD cells (WT BY4742 or *tsa1Δ*) were prepared anaerobically and Pyk1 activity determined under anaerobic conditions. Assays included 50 μM PEP and 12 mM FBP. *Asterisk* indicates significant difference (*p* = 0.008, Students paired two-sided *t* test). *D*, effect of the *tsa1Δ* mutation on Pyk1 specific activity. Assays were performed as for C, and activity normalized to Pyk1 abundance as detected by immunoblotting with anti-Pyk1 antibody. *E*, effect of DTT on Pyk1 activity of ZnD yeast. WT yeast (BY4742) was grown in LZM + 1 μM Zn, and cell extracts were prepared aerobically ± 10 mM DTT in the extraction buffer. Pyk1 activity was measured in the presence of 1 mM FBP and 1 mM PEP, ± 10 mM DTT. Activity was normalized to Pyk1 expression level as determined by immunoblot quantitation. *Asterisk* indicates significant difference (*p* = 0.02, Students paired two-sided *t* test). *F* and *G*, effect of *cdc19*^*S492A*^ on PEP accumulation. Yeast strains of the indicated genotypes (CWM313, 314, 327, and 329) were grown to log phase in LZM + 100 μM (F) or LZM + 1 μM (G) Zn. PEP was assayed in cell extracts as described in Experimental procedures. Columns indicate the average of three (for panels *B* and *G*) or four (panels *C*, *D*, *E*, and *F*) experimental replicates and error bars indicate ± 1 SD. Fba1, fructose-bisphosphate aldolase 1; FBP, fructose 1,6-bisphosphate; LZM, low Zn medium; PEP, phosphoenolpyruvate; Pyk1, pyruvate kinase isozyme 1; ZnD, Zn-deficient.

Why would the activity of pyruvate kinase impact the ability of a *tsa1Δ* mutant to tolerate Zn deficiency? Pyk1 acts at the end of glycolysis to convert PEP into pyruvate in an essentially irreversible reaction (Fig. 4*A*). Notably, some PEP is also diverted to the biosynthetic chorismate pathway for the production of essential aromatic amino acids (phenylalanine, tyrosine, and tryptophan). Glycolysis, and therefore PEP production, is a Zn-dependent process. Most obviously, the Zn-dependent enzyme FBP aldolase (Fba1) is an abundant Zn-binding protein in replete cells and accumulates to a high level as a Zn-free apoprotein in ZnD cells (5). Consistent with this observation, aldolase activity measured in extracts of ZnD *tsa1Δ* and WT cells dropped to 10% or less of the activity observed in ZnR cells (Fig. 4*B*). Thus, we hypothesized that Tsa1 negatively regulates Pyk1 to adapt to the low aldolase activity in ZnD cells and preserve the supply of scarce glycolytic intermediates for accessory biosynthetic pathways. Consistent with this idea, Tsa1 was previously reported to negatively regulate Pyk1 activity during the diauxic shift (the transition from glycolysis to gluconeogenesis) (43). Tsa1 physically interacted with Pyk1 *in vitro* and inhibited its activity in response to H_2_O_2_. However, it is not yet clear if Pyk1 activity is redox-regulated, or the exact mechanism by which Tsa1 downregulates its activity in response to the diauxic shift.

Our model predicted that Pyk1 activity would decrease in low Zn, and that this decrease would be dependent on Tsa1 activity. To test this prediction, we compared Pyk1 activity in extracts from ZnR and ZnD WT and *tsa1Δ* cells (Fig. 4*C*). Preparation of cell extracts aerobically led to spurious oxidation, as indicated by novel DTT-sensitive species with faster migration in SDS-PAGE (Fig. S3*A*). This oxidation occurred *in vitro* since it was prevented by including the thiol-alkylating agent N-ethylmaleimide in the lysis buffer. Consequently, to prevent *in vitro* oxidation from complicating the results, cell lysates were prepared and Pyk1 activity assayed under anaerobic conditions. When we measured Pyk1 activity normalized to total protein in WT cell extracts, Zn deficiency was associated with a significant decrease in total activity (Fig. 4*C*, *p* = 0.008). However, activity of *tsa1Δ* cells was not significantly affected by Zn deficiency. This effect of the *tsa1Δ* mutation was primarily due to elevated Pyk1 specific activity in ZnD cells (Fig. 4*D*). The effect of Zn on Pyk1 activity was at least partially due to the changes in Pyk1 redox state, because Pyk1 activity in ZnD cells was significantly increased by inclusion of DTT during preparation of protein extracts and in the assay itself (Fig. 4*E*, *p* = 0.02). Dependence of Pyk1 activity on reduced thiol groups was supported by the observation that N-ethylmaleimide treatment of extracts strongly inhibited activity (Fig. S3*B*). Thus, these results suggest that Tsa1 inhibits Pyk1 activity in ZnD cells, and that Pyk1 activity is redox-dependent.

Our model also predicted that the lower aldolase activity in ZnD cells would restrict PEP accumulation. Accordingly, PEP decreased 8-fold in ZnD *versus* ZnR WT cells (Fig. 4*F* *versus* Fig. 4*G*). As expected given their lower Pyk1 activity, ZnD *cdc19*^*S492A*^ cells accumulated approximately 3-fold more PEP than WT cells (Fig. 4*G*). Although we also expected that the *cdc19*^*S492A*^ mutation would increase PEP accumulation by ZnD *tsa1Δ* cells, no significant difference was observed. We suggest that in *cdc19*^*S492A*^ *tsa1Δ* cells, PEP spared by lower Pyk1 activity is rapidly consumed by other pathways, and as a consequence does not accumulate under steady-state conditions. In *TSA1* WT cells however, these results confirmed the prediction that PEP is depleted in ZnD conditions, and that the *cdc19*^*S492A*^ allele promotes PEP accumulation.

**Figure 5:**
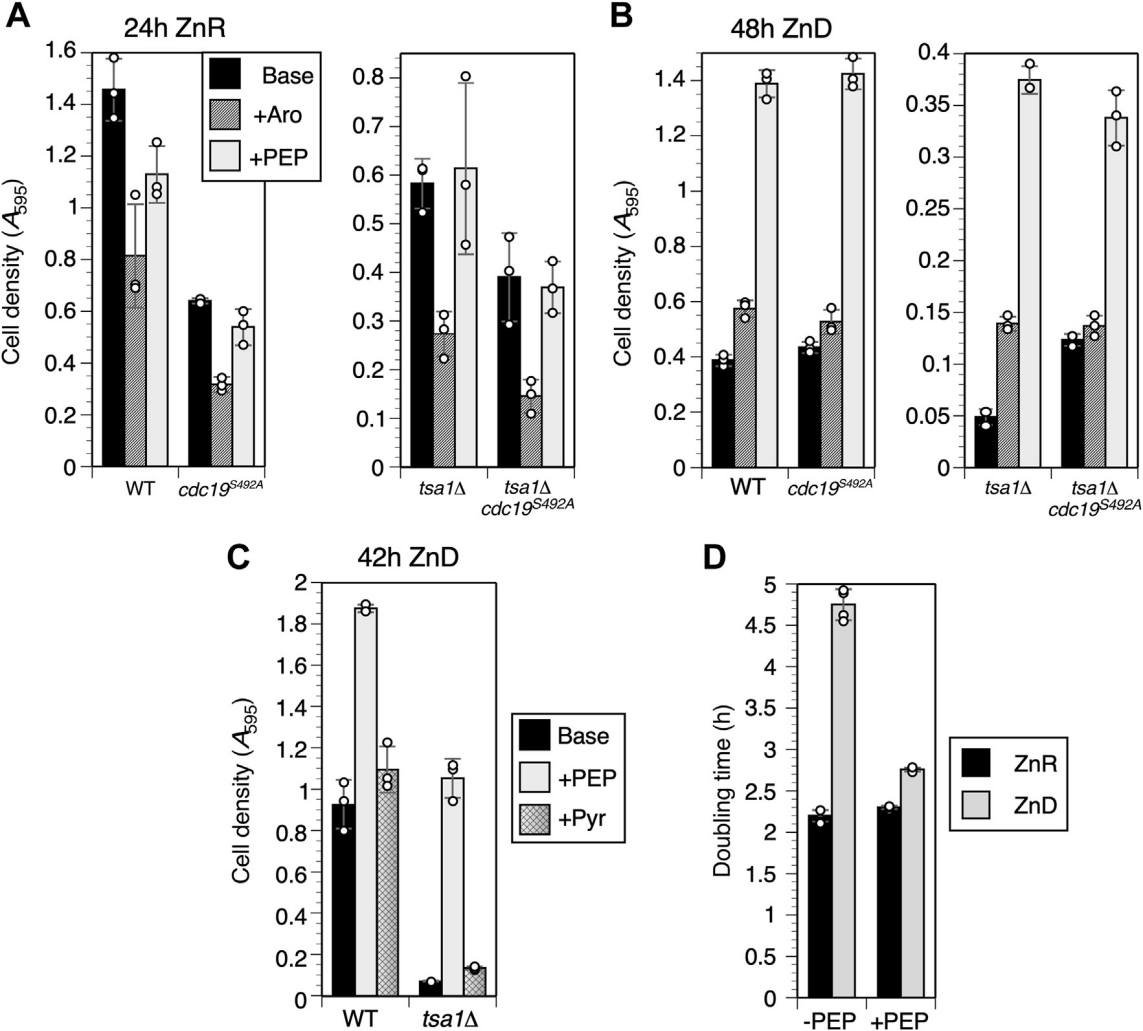
**Aromatic amino acid and PEP supplementation improve growth of ZnD cells.***A* and *B*, growth of fully prototrophic WT, *cdc19*^*S492A*^, *tsa1Δ* and double mutant strains (CWM313, 314, 327, and 329) after 24 h in LZM + 100 μM Zn (panel *A*) and 48 h in LZM + 1 μM Zn (panel *B*). Auxotrophic markers were corrected with the pHLK plasmid. Note *tsa1Δ* and double mutant data were plotted on separate scales for clarity. Base medium (LZM without auxotrophic supplements) was supplemented with the indicated compounds (+Aro = 0.01% phenylalanine, tyrosine, and tryptophan, +PEP = 0.5% phosphoenolpyruvate). Saturated SD cultures were used to inoculate LZM + 1 or 100 μM Zn to an *A*_595_ of 0.01 at the start of the experiment. *C*, comparison of the growth of WT and *tsa1Δ* mutant strains (CWM313 and 327 +pHLK) in minimal LZM + 1 μM Zn supplemented with 0.5% PEP or 0.5% pyruvate. The experiment was carried out as described for panel *A*. *D*, effect of 0.5% PEP supplementation on the doubling time of a WT prototrophic strain (CWM313 + pHLK) grown in LZM + 1 or 100 μM Zn. Columns indicate the average of three (panels *A*-*C*) or four (panel *D*) experimental replicates and error bars indicate ± 1 SD. LZM, low Zn medium; PEP, phosphoenolpyruvate; SD medium, synthetic defined medium; ZnD, Zn-deficient.

If Tsa1-mediated suppression of Pyk1 activity in ZnD cells and the *cdc19*^*S492A*^ mutation both conserve PEP in low Zn, we predicted that supplying biosynthetic products of PEP would improve growth of ZnD *tsa1Δ* cells. PEP is an essential precursor for the chorismate pathway, the sole source of the aromatic amino acids (44). To determine if ZnD cells displayed an aromatic amino acid auxotrophy, we tested the effect of additional tyrosine, tryptophan, and phenylalanine on growth of WT, *tsa1Δ*, *cdc19*^*S492A*^, and double mutant strains (Fig. 5). Supplementing ZnR cells with aromatic amino acids inhibited growth regardless of genotype (Fig. 5*A*). In contrast, growth of ZnD WT and *cdc19*^*S492A*^ cells was mildly improved by aromatic amino acids, and growth of ZnD *tsa1Δ* cells substantially improved (Fig. 5*B*). However supplementation had no effect on ZnD *tsa1Δ cdc19*^*S492A*^ cells (Figs. 5*B* and S4*D*). Aromatic amino acids essentially phenocopied the *cdc19*^*S492A*^ mutation, restoring *tsa1Δ* growth to the level of *tsa1Δ cdc19*^*S492A*^ cells. Consistent with their shared dependence on chorismate synthesis, addition of all three amino acids was required for this effect (Fig. S4*B*). These observations are consistent with the hypothesis that by suppressing Pyk1 activity in ZnD *tsa1Δ* cells, *cdc19*^*S492A*^ preserves PEP for aromatic amino acid synthesis. To identify other biosynthetic pathways that might be Tsa1 dependent, we tested all other amino acids for an effect on growth of ZnD WT and *tsa1Δ* cells (Fig. S4, *C* and *D*). Although some did promote growth (*e.g.,* aspartate and glutamate), these effects were not specific to ZnD cells. Thus, aromatic amino acids are unique in their ability to specifically improve the slow growth of ZnD *tsa1Δ* cells.

If the aromatic amino acid auxotrophy we observed in *tsa1Δ* was a direct consequence of PEP depletion, we anticipated that supplementing with PEP would also specifically correct this auxotrophy in ZnD *tsa1Δ* mutant cells. Remarkably, however, ZnD WT, *tsa1Δ*, *cdc19*^*S492A*^, and double mutant strains all showed major increases in growth when supplemented with PEP (Fig. 5*B*). This effect was specific to ZnD cells, as supplementing ZnR cultures had no effect or inhibited growth (Fig. 5*A*). Because excess PEP could be converted to pyruvate by Pyk1, it was possible that pyruvate was the growth-promoting factor. However, pyruvate supplementation had little effect on growth of ZnD cells (Fig. 5*C*). To more directly compare the effect of PEP on growth of ZnD and ZnR cells, we determined doubling times during growth of WT cells under these conditions. WT cells in a ZnR medium grew with a rate of 2.2 ± 0.07 h/doubling while ZnD WT cells had a growth rate of 4.75 ± 0.19 h/doubling (Fig. 5*D*). Adding PEP to the medium had no significant effect on ZnR WT cells but accelerated the growth rate of ZnD cells to 2.76 ± 0.02 h/doubling. Thus, PEP supplementation of ZnD WT cells accelerated growth to nearly the rate of ZnR cells. We conclude that disrupted glycolysis and the resultant PEP deficiency is a major reason why WT ZnD yeast cells grow slower than the replete cells.

**Figure 6:**
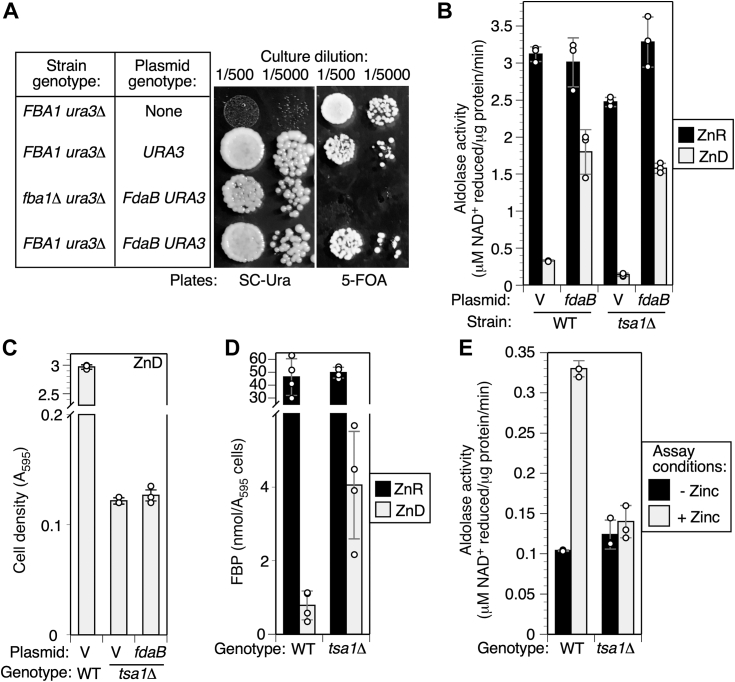
**Aldolase activity is not the major limit on glycolysis in ZnD cells.***A*, FdaB can functionally substitute for the essential aldolase Fba1. A WT strain with or without a *URA3* control vector (BY4742 ± pFL44-S), and an isogenic *fba1Δ* strain (CWM370) with a *URA3* vector carrying FdaB (pFdaB) were grown to saturation in SC medium to allow plasmid loss. Cultures were diluted as indicated and 10 μl applied to either SC-uracil plates or 5-FOA plates (a negative selection for the *URA3* gene). *B*, *fdaB* expression increased aldolase activity in ZnD cells. Cultures of the indicated strains (BY4742 and BY4742/*tsa1Δ* carrying empty vector pFL38 or pFdaB plasmids) were grown to log phase in LZM + 1 or 100 μM Zn. Aldolase activity was measured in cell extracts as described in Experimental procedures. To ensure full activity of Fba1 aldolase, assays were supplemented with Zn (5 μM). *C*, *fdaB* expression and elevated aldolase activity did not suppress the *tsa1Δ* growth defect in ZnD yeast. Cultures of the indicated strains were inoculated to 0.01 starting *A*_595_ in LZM + 1 μM Zn and grown for 3 days before recording final *A*_595_. *D*, FBP levels were substantially reduced in ZnD cells, but elevated in ZnD *tsa1Δ* cells. WT (BY4742) and *tsa1Δ* cultures were grown to log phase in LZM + 100 μM (ZnR) or 1 μM (ZnD) Zn, harvested by cold methanol quench, and metabolites extracted with perchloric acid. FBP was measured using a commercial assay kit. *E*, aldolase from *tsa1Δ* ZnD cells is insensitive to activation by Zn *in vitro*. WT BY4742 and *tsa1Δ* strains were grown to log phase in LZM + 1 μM Zn, and activity was assayed ± 5 μM Zn in the assay buffer. Columns indicate the average of three (panels *B*, *C*, and *E*) or four (panel *D*) experimental replicates and error bars indicate ± 1 SD. Fba1, fructose-bisphosphate aldolase 1; FBP, fructose 1,6-bisphosphate; FOA, 5-fluoroorotic acid; LZM, low Zn medium; SC, synthetic complete; ZnD, Zn-deficient; ZnR, Zn-replete.

Our hypothesis of disrupted glycolysis in Zn deficiency was based on the recognition that Fba1 aldolase is a Zn-dependent enzyme with substantially lower specific activity in ZnD cells (Fig. 4*B*) (5). To test this prediction, we expressed a Zn-independent aldolase enzyme in yeast, FdaB from *Staphylococcus aureus* (45). As an initial test of FdaB function in yeast, we examined its ability to substitute for the essential Fba1 protein. Starting with a haploid strain carrying the *fdaB* gene on a *URA3* plasmid, we deleted *FBA1* from the yeast genome. This experiment generated a viable *fba1Δ::kanMX4* mutant that could not grow on medium counter selective for *URA3* function, indicating that it was absolutely dependent on the plasmid-borne FdaB gene for growth. Thus FdaB could functionally replace Fba1 (Fig. 6*A*).

We then measured total aldolase activity in *FBA1 TSA1* (WT) and *FBA1 tsa1Δ* strains carrying the FdaB plasmid or vector alone. In these experiments, assays were supplemented with Zn to ensure metalation of all active Fba1 (omission of Zn inhibited Fba1 activity, Fig. S5). Extracts of ZnR cells contained abundant aldolase activity regardless of genotype (Fig. 6*B*). However, extracts from ZnD WT vector-only cells had approximately 10-fold less activity. This is consistent with our previous observations that ZnD cells accumulate inactive Fba1 apoprotein that is insensitive to reactivation by Zn (5). When FdaB was expressed in ZnD cells, aldolase activity increased, reaching approximately 50% of the activity observed in ZnR cells. FdaB expression also restored a similar level of aldolase activity to ZnD *tsa1Δ* cells. Despite this high level of Zn-independent aldolase activity, however, *tsa1Δ* cells expressing FdaB grew no better than vector-only control cells in low Zn (Fig. 6*C*). Together, these results argue that aldolase activity is not the only factor limiting glycolysis in low Zn. Our hypothesis also predicted that the decreased activity of Fba1 aldolase in ZnD cells would promote FBP accumulation when compared to replete cells. However, we found that FBP levels were greatly reduced in ZnD *versus* ZnR WT and *tsa1Δ* cells (Fig. 6*D*), further indicating that in ZnD cells, glycolysis is restricted at one or more steps upstream of Fba1 aldolase.

## Discussion

In this report, we describe our analysis of a suppressor mutation that partially reversed the growth defect of *tsa1Δ* mutants in ZnD conditions. During this analysis, we made the unexpected discovery that in both *tsa1Δ* and WT cells, glycolysis is severely disrupted by Zn limitation at an early step in the pathway. This result was surprising because hundreds of yeast proteins require Zn for function, and many of these are essential for growth and viability. While loss of function of any one of these proteins might be expected to slow growth, our observations suggest that the majority of the growth defect in Zn deficiency can be attributed to just a single issue, impaired glycolysis.

The discovery that glycolysis is a critical vulnerability for yeast in low Zn is extremely valuable because these results identify an “Achilles heel” for pathogenic fungi. Vertebrates fight microbial infections in part by depriving invading cells of essential metals ("nutritional immunity"), *e.g.* by binding and sequestering Zn and manganese with antimicrobial proteins like calprotectin. Consumption of glucose by fungal and bacterial pathogens is essential during infection (46), and, in most microbes, glycolysis is Zn dependent due to the utilization of Class II (metal-dependent) FBP aldolases (although Mn-dependent enzymes have been described, canonical class II aldolases are Zn-dependent) (45). Some pathogens have mechanisms to minimize their Zn requirement during infection. *S. aureus*, for example, has two aldolase genes, one encoding a Mn-dependent aldolase (FbaA) and the second a metal-independent aldolase (FdaB). FdaB functionally replaces FbaA during infection and confers resistance to calprotectin (45). Despite this finding, however, it appears that relatively few microbes have metal-independent aldolase enzymes, suggesting aldolase and glycolysis are prime targets for mechanisms of nutritional immunity (47).

In this work, we made the surprising discovery that in addition to the strongly negative effect of Zn deficiency on Fba1 aldolase activity, glycolysis is also restricted at one or more steps upstream of Fba1. Therefore, glycolysis represents a critical process that is extremely vulnerable to Zn restriction. If this finding proves generally true for other fungi and microbes, therapeutic treatments designed to further impair fungal glycolysis would likely be valuable additions to the arsenal of tools that are available to fight fungal infections. It is unclear at this time where the specific defect in glycolysis lies. We initially considered that the loss of activity of Zn-dependent FBP aldolase was a likely explanation. This hypothesis was attractive because it predicted that Fba1 deficiency would elevate FBP accumulation, which in turn should allosterically activate Pyk1, further depleting PEP (Fig. 4*A*). However, FBP levels were nearly 50-fold lower in ZnD WT cells, suggesting aldolase activity is not limiting. In addition, expression of a Zn-independent aldolase, FdaB, did not suppress the growth defect of a *tsa1Δ* mutant as expected. Thus, although impaired FBP aldolase activity may contribute to decreased glycolytic flux overall, our observations indicate that the primary defect in glycolysis arises from one or more Zn-dependent events upstream of FBP aldolase. The three steps in the pathway from glucose to FBP are catalyzed by hexokinase (encoded in yeast by *HXK1*, *HXK2*, and *GLK1*), glucose 6-phosphate isomerase (encoded by *PGI1*) and phosphofructokinase (encoded by *PFK1* and *PFK2*). None of these enzymes are known to require Zn for function. Zn might be required indirectly for their accumulation; however, our previous survey of the effect of Zn deficiency on the proteome (5) revealed either no major changes in glycolytic enzymes (*e.g.* Pgi1, Pfk1, and others) or their induction (*e.g.* Hxk1, Glk1, and others) (Fig. S6, *A* and *B*). This general insensitivity of glycolytic enzyme abundance to zinc deficiency (with some notable exceptions, see below) also appears to exclude possible inhibition of Zn-dependent transcriptional or translational machinery as an explanation for glycolytic inefficiency.

Our previous studies (5) also argue for the importance of glycolytic adaptation to low Zn conditions. The genes encoding two glycolytic enzymes (*TDH1* and *ENO1,* encoding isozymes of glyceraldehyde-3-phosphate dehydrogenase and enolase) are targets of Zap1 transcriptional regulation (16) and these proteins were strongly induced in ZnD cells as expected (Fig. S6*B*). Although the effect of this induction on pathway activity is unknown, our observations here suggest that these isozymes may facilitate adaptation of the second phase of glycolysis to low Zn. More unexpectedly, the hexokinases Hxk1 and Glk1 were also strongly induced. The genes encoding these proteins are not Zap1 targets, and are normally repressed in glucose-grown cells, where Hxk2 is the dominant hexokinase isozyme (48). A switch from Hxk2 expression to Hxk1 and Glk1 is characteristic of cells supplied with non-fermentable carbon sources. The strong induction of these proteins in low Zn suggests that ZnD cells are defective for glucose sensing or uptake, and induce a regulatory response with characteristics of glucose derepression. Consistent with this interpretation, high affinity hexose transporters Hxt6 and 7 were induced in low Zn, as were enzymes of trehalose metabolism (Tps1, Tps2, and Tsl1), and glycogen synthesis (Gsy1, Gsy2, and Gph1) (Fig. S6, *B* and *C*). All of these enzymes are normally repressed in high glucose (49, 50), suggesting ZnD yeast sense glucose limitation despite an abundance of glucose in the medium. However, the same proteomic experiment did not detect induction of the gluconeogenesis enzymes Pck1 or Fbp1, suggesting that the apparent glucose limitation is not severe enough to induce gluconeogenesis. More work is required to describe the specific features of this newly identified glycolytic adaptation to low Zn and identify how it differs from the currently understood regulatory response to glucose depletion (51).

The identification of the Pyk1^S492A^ mutation as a suppressor of the *tsa1Δ* growth defect suggested that Tsa1 might downregulate pyruvate kinase to facilitate adaptation to low Zn. In agreement with this idea, a previous group reported that Tsa1 downregulated Pyk1 activity during the diauxic shift, the period during which cells deplete glucose and switch to metabolizing the ethanol produced by fermentative growth (43). Gluconeogenesis utilizes PEP carboxykinase to convert oxaloacetate into PEP, and it was suggested that the inhibition of Pyk1 by Tsa1 helped prevent the futile cycle that would occur if the PEP generated by PEP carboxykinase was immediately converted back into pyruvate by Pyk1. Why might ZnD cells supplied with abundant glucose decrease Pyk1 activity? As previously mentioned, we detected no induction of gluconeogenesis enzymes in low Zn, arguing against significant futile cycling. Instead, we propose that Pyk1 is inhibited by Tsa1 to adapt to lower glycolytic flux by conserving scarce glycolytic intermediates such as PEP, allowing them to be diverted for synthesis of aromatic amino acids and possibly other compounds. How might Tsa1 inhibit Pyk1 in ZnD cells? We previously detected increased ROS/RNS species in ZnD yeast using the reagent 2,7-dichlorodihydrofluorescein (23). Although the identity of the specific ROS/RNS detected by 2,7-dichlorodihydrofluorescein *in vivo* is controversial (52, 53, 54, 55), it is possible that the Tsa1 substrates H_2_O_2_, lipid hydroperoxides, or peroxinitrite contribute to this signal. We hypothesize that in ZnD cells, these elevated ROS/RNS constitute a signal for low Zn that is sensed by Tsa1, which in turn oxidizes Pyk1. Our data showing redox-sensitive activity of Pyk1 are consistent with this model, although the details of the regulatory mechanism remain to be described.

A possible criticism of this model is that the magnitude of the Tsa1-dependent inhibition of Pyk1 was relatively moderate, and was much less than the effect of the S492A mutation. Assuming that *in vitro* assays of Pyk1 activity do not underestimate the magnitude of Tsa1-mediated inhibition *in vivo*, it is not clear if the moderate Tsa1-dependent inhibition we observed would restrict Pyk1 activity enough to restore aromatic amino acid synthesis. One possible explanation is that Tsa1 may regulate PEP availability not just via Pyk1 activity, but also at other points in glycolysis. For example, our data suggest that Tsa1 positively regulates FBP aldolase activity in ZnD cells. The activity of aldolase isolated from ZnD WT cells was increased by adding Zn to the assays, suggesting that a fraction of aldolase apoprotein could productively bind its cofactor *in vitro* (Fig. 6*E*). However, Zn addition had no effect on activity of aldolase isolated from ZnD *tsa1Δ* cells. These observations suggest that Tsa1 can protect apo-aldolase from irreversible inactivation. Although the amount of activity that was protected by Tsa1 was relatively small, in the context of the overall aldolase limitation, this small difference might affect fitness. The observation that FBP levels were higher in ZnD *tsa1Δ* cells than WT cells (Fig. 6*D*) is also consistent with Tsa1 enhancing aldolase activity *in vivo*. It is interesting to speculate that the protein chaperone activity of Tsa1 might play a role in its support of Fba1 activity, perhaps by stabilizing the apoprotein (20). However, the mechanisms underlying these effects remain to be explored. Thus, Fba1 represents a novel potential target for Tsa1 regulation, and other targets may also exist. A comprehensive survey of Tsa1-interacting proteins is required to understand the full effect of Tsa1 on glycolysis under ZnD conditions.

A major question that arises from this work is why PEP supplementation so effectively aided the growth of ZnD cells. The observation that PEP boosted growth of all strains tested, but that the effect of aromatic amino acids was more moderate and specific to *tsa1Δ* mutants (Fig. 5*B*), indicates that PEP deficiency also limits another essential process in ZnD cells. Obvious candidates include other amino acid synthesis pathways that are dependent on glycolytic intermediates upstream of PEP. For example, in glucose-grown cells serine and glycine biosynthesis is primarily initiated by the action of 3-phosphoglycerate dehydrogenase (Ser3/33) on the glycolytic intermediate 3-phospho-D-glycerate (56). However, we observed no effect on growth from supplementing ZnD WT or *tsa1Δ* cells with serine or glycine (Fig. S4), suggesting this pathway is unaffected by PEP deficiency. This observation may reflect the presence of alternative pathways for serine synthesis that potentially substitute for glycolysis-dependent synthesis in ZnD cells (57, 58, 59). A second and perhaps more exciting explanation for the strong effect of PEP on growth of ZnD cells was suggested by recent work showing that Pyk1 moonlights as a protein kinase with regulatory functions. In yeast, Pyk1 forms part of the serine-responsive SAM-containing metabolic enzyme complex (SESAME) (60) which is involved in autoregulating *CDC19* expression. This function requires Pyk1 to phosphorylate histone H3 at threonine residue 11, and is promoted by abundant supply of PEP and FBP from glycolysis (61). This function of yeast Pyk1 is directly recapitulated by mammalian PKM2 (62). PKM2 is a minor isozyme of pyruvate kinase in vertebrates, but is unregulated in many cancer cells. Its expression is associated with the Warburg effect, or the fermentation of glucose to lactate under aerobic conditions, a major characteristic of cancer cells (63). The importance of PKM2 kinase function was enhanced by the discovery that phosphoribosylaminoimidazolesuccinocarboxamide (SAICAR), an abundant metabolite in proliferating cells, activates this protein kinase activity (64). This discovery allowed the identification of hundreds of novel targets for the PKM2 protein kinase, many of which are themselves kinases important for cell proliferation. These findings suggest that supplying PEP to ZnD yeast may support cell growth by supplying Pyk1 with the substrate necessary for its protein kinase activity (61), allowing the activation of cell proliferation pathways normally suppressed by PEP deficiency.

Regardless of how PEP aids growth of ZnD cells, to our knowledge this is the first study to identify a single specific effect of Zn deficiency, glycolytic inefficiency, as a major contributor to the slow growth of eukaryotic cells. Our discovery provides a surprising insight into the effect of Zn deficiency on fitness, with important implications both for the development of interventions to combat fungal disease, and for our general understanding of the microbial response to Zn deficiency.

## Experimental procedures

### Yeast strains and growth media

Yeast strains were routinely grown in yeast extract peptone dextrose medium with 2% glucose or 3% glycerol, 2% ethanol (YPGE), synthetic defined medium, or low Zn medium (LZM) as described previously (20, 21). LZM lacks added Zn and contains chelators (EDTA and citrate) to reduce Zn availability. LZM was supplemented with 1 or 100 μM ZnCl_2_ to generate ZnD or replete conditions, respectively. To aid growth of S288c-derived strains, LZM was routinely supplemented with a subset of amino acids and inositol (65), with the exception of experiments testing the effect of such supplements on growth.

### Yeast strain construction

Yeast strains used in this work are listed in Table S2. All yeast strains are isogenic to the haploid BY4741 and BY4742 strains (66). Deletion mutant strains from the BY4743 collection or viable BY4742 collection were obtained from Invitrogen. Additional multiply marked mutant strains were generally derived from single mutants via transformation with KO constructs, and their correct modification verified with PCR. As Pyk1 is essential for growth on fermentable carbon sources such as glucose, *cdc19* deletion mutant strains were generated and propagated on yeast extract peptone medium plates with 3% glycerol and 2% ethanol (YPGE). To generate a haploid *cdc19Δ::kanMX4* deletion mutant, the *cdc19Δ::kanMX4* marker was amplified with PCR from genomic DNA of a heterozygous diploid strain (Invitrogen) (67) using the oligonucleotides pyk5′kanamp (5′-CATGGCAACGTCACCTCA-3′) and 3′pyk1seq (5′-TGCGATGGGAGGGATCAA-3′) and used to transform BY4742. Cells were plated on YPGE + G418 to select for the mutant (CWM307). Introduction of plasmids into CWM307 was achieved by growth in YPGE medium, transformation using the LiOAc/PEG method, outgrowing transformed cells in YPGE liquid medium for 12 h, and plating cells on yeast extract peptone dextrose to select for complemented strains. This process was used to generate all plasmid-bearing *cdc19Δ::kanMX4* strains (CWM313, 314, 331, and 333), which were then used to generate their *tsa1Δ* mutant derivatives (CWM327, 329, 337, and 339) via transformation with a *tsa1Δ::LEU2* PCR product as previously described (20). To generate CWM370, which expresses bacterial FdaB in an *fba1Δ* background, the pFdaB plasmid was first introduced into BY4742, then *FBA1* was deleted by introduction of a PCR product of the *fba1Δ::kanMX4* marker amplified from genomic DNA of a heterozygous *fba1Δ::kanMX4* diploid (Invitrogen) (67) using the oligonucleotides fbamut (5′-AATGACTCCGCAGTGGAC-3′) and fba15′ (5-TGACGCAAGCCCTAAGAA-3′).

### Plasmid construction

Plasmids used in this work are listed in Table S3. pRS313-HA-PYK1 was supplied by Shusuke Kuge (43), and the p413TEF-PYK1 and p413TEF-PYK2 plasmids by Markus Ralser (42). All plasmids constructed for this work were completely sequenced using an Oxford Nanopore GridION Mk1 and sequences are available upon request. To construct p*CDC19* and p*cdc19*^*S492A*^, a 3.3 kb fragment containing the *CDC19* gene was amplified from genomic DNA of WT BY4742 or a *cdc19*^*S492A*^ mutant (strain 23-1D/22), using the oligonucleotides 5′pyk1 (5′-CACGACGTTGTAAAACGACGGCCAGTGAATTCGCAGTTGTCTGCGGGCAC-3′) and 3′pyk1 (5′-GAAACAGCTATGACCATGATTACGCCAAGCTTAATCTGCCCGCGTCATTT-3′). pFL38 vector digested with *Eco*RI and *Hind*III was combined with the PCR product and used to transform BY4742 selecting *URA3*^+^. Plasmid DNA was rescued from transformants and the plasmid insert verified by sequencing.

The plasmid pFdaB was derived by combining three PCR fragments with pFL44-S (68) by homologous recombination in yeast. A 547 bp fragment of the *PGK1* promotor was amplified from genomic DNA with the primers YNL-PGK1 5′ (5′-ACGACGTTGTAAAACGACGGCCAGTGAATTCTTAGATTCCTGACTTCAAC-3′) and PGK-Fda3′ (5′-CATTTTTCATTTTTTCTAATTGCTCTTTATTAGACATTGTTTTATATTTGTTGTA-3′). The FdaB coding sequence was amplified from a plasmid clone (45) using the primers fdaB5′ (5′-GAAGTAATTATCTACTTTTTACAACAAATATAAAACAATGTCTAATAAAGAGCAATTAGAAAAAATGAAAAATGGAAAAGG-3′) and fdaB3′ (5′-TTCAAAAAAATAATATCTTCATTCAATCATGATTCTTTTTTTAGTTTTTGTTTACAGATGCGTC-3′). The Pyk1 terminator was amplified from BY4742 genomic DNA using the primers pyk1term5′ (5′-CAATCTACGACGCATCTGTAAACAAAAACTAAAAAAAGAATCATGATTGAATGAAGAT-3′) and 3′pyk1-n (5′-GAAACAGCTATGACCATGATTACGCCAAGCTTAATCTGCCCGCGTCATTT-3′). The pFL44-S vector was digested with *Eco*RI and *Hind*III, and the three fragments combined with the cut vector for yeast transformation followed by selection of *URA3*^*+*^ clones. Plasmid DNA was rescued from transformants and sequenced to verify. Function of the resulting clones in yeast was verified by complementation of the *fba1Δ* mutation as described in the Results section.

### Pooled linkage analysis and whole genome sequencing

The screen that identified *cdc19*^*S492A*^ was previously described in detail (20). Briefly, we took advantage of the mutator phenotype of a *tsa1Δ* strain (CWY8) (23) to generate novel mutations during sequential rounds of selection for growth under ZnD conditions. From cultures showing robust growth, individual mutant strains were isolated and the nature and inheritance of the mutations was characterized by backcrossing to an isogenic *tsa1* mutant strain and sorting into complementation groups. One strain (23-1D) represented a new complementation group, and the responsible mutation was identified by pooled linkage analysis and WGS as previously described (20).

### Protein extraction and immunoblotting

Protein extraction with trichloroacetic acid, immunoblotting, and protein detection by immunoblotting using a Li-Cor Odyssey infrared dye detection system were performed as previously described (20). Protein abundance was quantified from immunoblots using Image Studio version 5.2.5 (LiCor) (licor.com/bio/image-studio/). Rabbit anti-Pyk1 antibody (69) was a gift of Jeremy Thorner, and rabbit anti-Fba1 antibody (70) a gift of Magdalena Boguta. Anti-Pgk1 (22C5D8) was obtained from Abcam. IRDye^R^ 680LT dye-labeled anti-mouse antibody and IRDye^R^ 800CW dye-labeled anti-rabbit antibody were obtained from Li-Cor. Antibody specificity in immunoblots was verified by comparison of GFP-tagged and untagged target molecular weight via immunoblotting, and/or by comparison of protein samples from WT and deletion mutant strains.

### Enzyme assays

β-galactosidase activity in yeast cells and fructose-1, 6BP aldolase activity in cell lysates were assayed as previously described (5, 20). Aldolase assays contained 1 to 5 μg of yeast lysate. Pyruvate kinase activity was determined in yeast extracts using a coupled assay (5). Briefly, cells grown in LZM + 1 or 100 μM ZnCl_2_ were collected and resuspended in an extraction buffer containing 20 mM Hepes (pH 7.1), 100 mM KCl, 1× fungal protease inhibitor cocktail (Sigma-Aldrich P8215), 1 mM PMSF, 5 mM Na_2_-EDTA, and 5 μM MG132. Cells were lysed by vortexing with 0.5 μm glass beads for 10 min. After clarification by centrifugation (12,000*g*, 15 min, 4 °C), supernatants were collected and protein concentration assayed using Bradford reagent (Bio-Rad) with bovine serum albumin as standard. Assays were performed by adding yeast lysate (5 μl, 0.25 μg) to wells of a 96-well plate, and the reaction started by adding an aliquot of assay mixture (25 mM potassium phosphate pH 7.6, 10 mM MgCl_2_, 5 mM ADP, 0.2 mM NADH, 1 U/ml rabbit lactate dehydrogenase [Sigma-Aldrich L-2500], and 0.5 mM PEP). Standard assays contained 1 mM FBP to activate PK, but PEP and FBP concentrations were varied as required for the experiment. Progress of the reaction was continuously monitored by the decrease in NADH absorbance at 340 nm. The maximum rate was calculated from the linear portion of the curve and converted to values of nmole NADH consumed/μg protein/minute using a standard curve of *A*_340_ *versus* NADH concentration. To determine specific activity, Pyk1 abundance in the samples was quantified by immunoblotting and rates normalized to those values. To measure Pyk1 activity in anaerobic conditions, yeast cells were grown and harvested as normal, then cell pellets were flushed with nitrogen and allowed to equilibrate in an anaerobic chamber for 2 h before extraction. Extractions and assays were performed with reagents flushed with nitrogen and stored anaerobically for at least 24 h.

### Metabolite assays

To preserve the *in vivo* state of metabolites, PEP and FBP were assayed in extracts prepared using cold methanol quenching (71) and perchloric acid extraction (72). Yeast LZM cultures (15 ml) were grown to log phase and harvested by addition to 30 ml methanol prechilled to −80 °C in a dry ice/ethanol bath. The mixture was chilled back to −80 °C on dry ice, and cells were rapidly collected by centrifugation in a 50 ml tube rotor insert chilled to −80 °C (4000 rpm/1 min). The medium was discarded, and cells were resuspended in the residual liquid and transferred to 1.5 ml centrifuge tubes held at −80 °C. Cell pellets were rapidly collected by centrifugation in a microfuge rotor chilled to below −30 °C with dry ice, and stored frozen at −80 °C or immediately processed with perchloric acid. To extract metabolites, 200 μl of ice cold perchloric acid (3 M) was added to the frozen cell pellet and the mixture thawed on ice with repeated vortexing. A 0.2 ml volume of glass beads (425–600 mesh size, Sigma-Aldrich) was added and the tubes were vortexed at 4 °C for 10 min. The mixture was then neutralized by sequential addition of 10 μl aliquots of 3 M potassium bicarbonate, monitoring pH by addition of 1 μl aliquots to pH indicator strips. When pH reached 7.0, insoluble material was removed by centrifugation at 16,000*g*/0 °C for 5 min, and the supernatant metabolite solutions were stored at −80 or used immediately to assay PEP and FBP. PEP was assayed by colorimetric detection using a commercial kit (MAK102, Sigma-Aldrich) following the manufacturer’s instructions. Background pyruvate signal (from control reactions that did not convert PEP to pyruvate) was subtracted, and absolute values of PEP in the samples were determined by comparison with a standard curve. FBP was assayed using a fluorometric kit (ab284537, Abcam) following the manufacturer’s instructions.

## Data availability

All data generated in this study are available by request to C. M.

## Supporting information

This article contains supporting information (73, 74).

## Conflict of interest

The authors declare that they have no conflicts of interest with the contents of this article.
