# Supplementary material for: Restricted glycolysis is a primary cause of the reduced growth rate of zinc-deficient yeast cells
Source: J Biol Chem. 2024 Mar 7;300(4):107147. doi: 10.1016/j.jbc.2024.107147 (PMC11001634; doi:10.1016/j.jbc.2024.107147)
Supplement: Supporting Figures S1–S6 and Table S1–S3 [file mmc1.docx]

**Supporting information**

**Restricted glycolysis is a primary cause of the reduced growth rate of**

**zinc-deficient yeast cells**

Colin W. MacDiarmid, Janet Taggart, Michael Kubisiak, and David J. Eide

**Contents:**

Supplemental Figures S1-S6

Table S1 Single nucleotide polymorphisms unique to the *stz3* gamete pool

Table S2 Yeast strains used

Table S3 Plasmids used


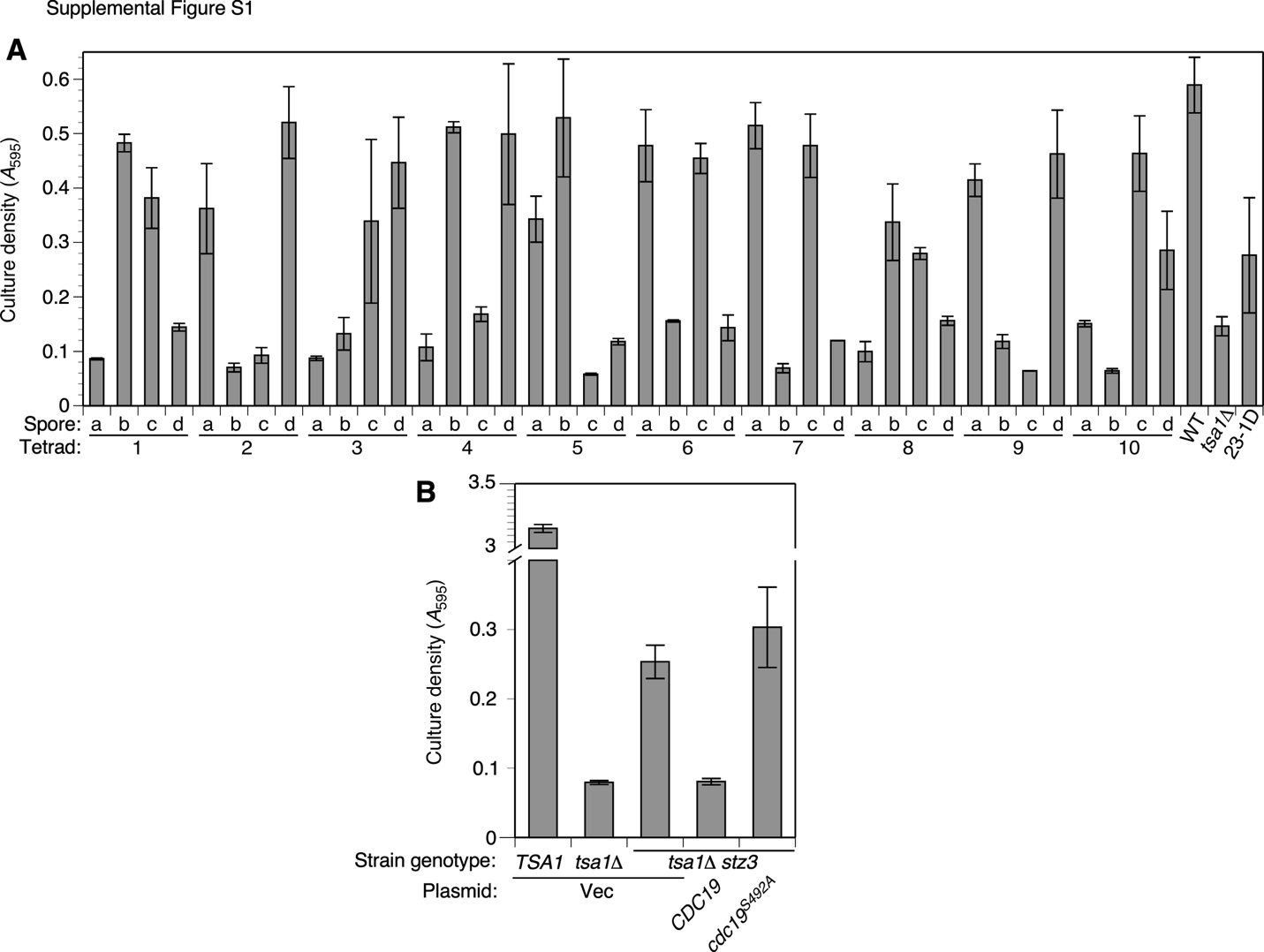


**Supplemental Figure S1:** **Identification of *stz3*.**

A) Full set of growth data for 40 spore clones from 10 tetrads derived from sporulation of a 23-1D/22 x CWY8 diploid (genotype *tsa1Δ/tsa1Δ* *CDC19/cdc19^S492A^*) in comparison with control strains CWY2 (*TSA1*), CWY8 (*tsa1Δ*) and the original 23-1D suppressed strain. Cultures were inoculated to an initial *A*_595_ of 0.01 in LZM with 1 μM zinc and grown for 3 days. B) Complementation of the *stz3* phenotype by wild-type *CDC19*. Wild-type (CWY2), *tsa1Δ* (CWY8) and *tsa1Δ* *stz3* (23-1D) strains transformed with the indicated plasmids (empty vector pFL38, pCDC19, or pcdc19^S492A^) were grown for three days in ZnD medium as described in A. All values are the average of three experimental replicates and error bars indicate ± 1 SD.


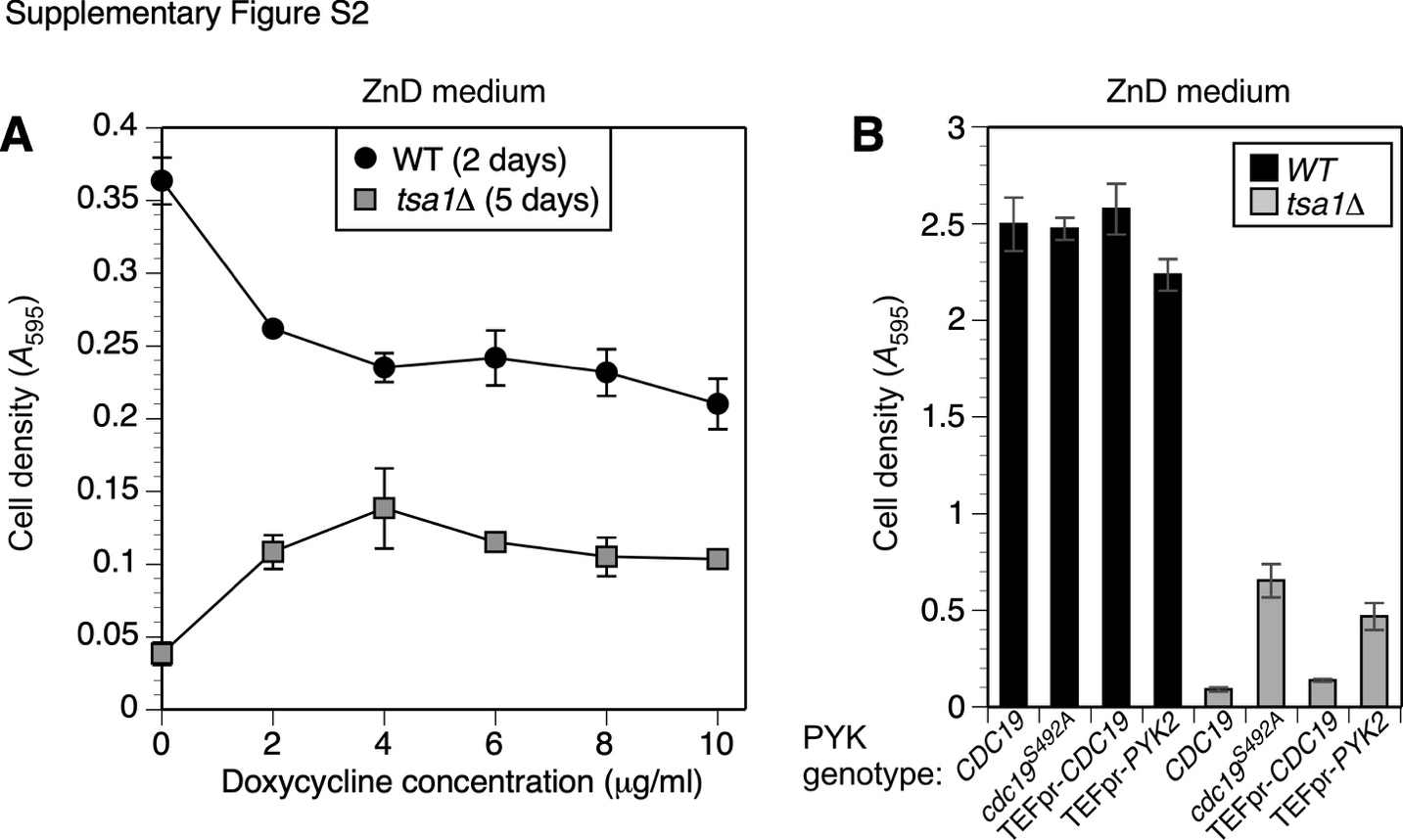


**Supplemental Figure S2. Lowering wild-type pyruvate kinase activity suppressed *tsa1Δ.***

A) Reduced expression of wild-type *CDC19* slowed growth of wild-type *TSA1* strains in low zinc, but increased growth of *tsa1Δ*. Growth of wild-type or *tsa1Δ* mutant strains carrying a *CDC19* allele (*cdc19^TetO7^*) repressible by addition of doxycycline. Strains were grown to saturation in SD medium, and then used to inoculate zinc deficient medium (LZM + 1 μM zinc with the indicated concentration of doxycycline) to an initial *A*_595_ of 0.01. Cultures were grown for 2 days (for WT) or 5 days (for *tsa1Δ*) before measurement of cell density. Yeast strains used were TH4015 (*TSA1* *cdc19^TetO7^*) and CMW343 (*tsa1Δ cdc19^TetO7^*). B) Substitution of *CDC19* with *PYK2* suppressed *tsa1Δ*. Wild-type or *tsa1Δ* strains expressing *CDC19* or *PYK2* from plasmids were used to inoculate zinc deficient medium to an initial *A*_595_ of 0.01 and cultures were grown for four days. Yeast strains were CWM313 (*TSA1 CDC19*), CWM314 (*TSA1* *cdc19^S492A^*), CWM331 (*TSA1* TEFpr-*CDC19*), CWM333 (*TSA1* TEFpr-*PYK2*), CWM337 (*tsa1Δ* TEFpr-*CDC19*) and CWM339 (*tsa1Δ* TEFpr-*PYK2*). All values are the average of three experimental replicates and error bars indicate ± 1 SD.


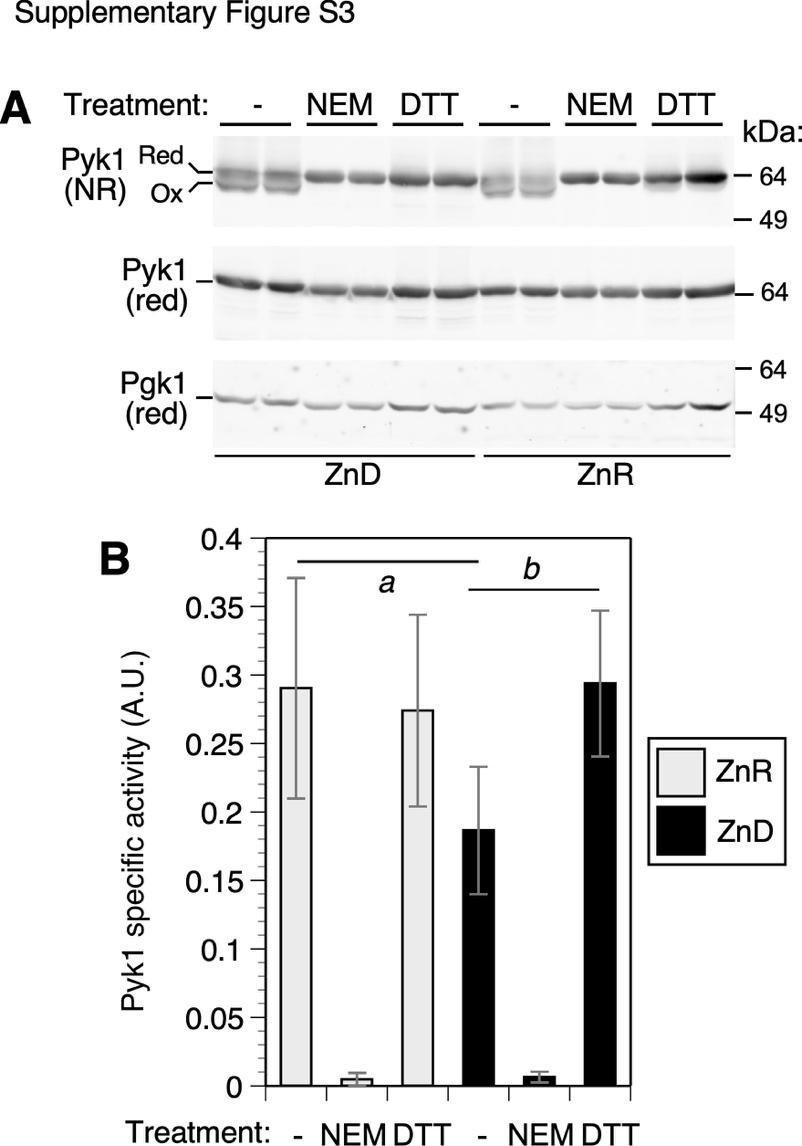


**Supplemental Figure S3. Pyk1 activity is thiol dependent.**

A) Oxidation of Pyk1 during protein extraction under aerobic conditions. Immunoblot of native protein extracts from a wild-type yeast (BY4742), prepared using extraction buffer with no addition (-), 10 mM NEM, or 10 mM DTT. SDS-PAGE loading buffer without (NR) or with (red) 0.1 M DTT was added to samples prior to SDS-PAGE, and immunoblotting was performed with anti-Pyk1 and anti-Pgk1 antibodies. Two experimental replicate samples are shown for each condition. B) Pyk1 activity is sensitive to thiol oxidation and modification. Pyk1 activity in aerobically-prepared samples was assayed in aerobic conditions and normalized to Pyk1 abundance as determined by immunoblotting. Data are the average of four experimental replicates and error bars indicate ± 1 S.D. Significant differences between comparisons are indicated (*p* = 0.024 for *a*, 0.02 for *b*, Students paired two-sided t-test).

**
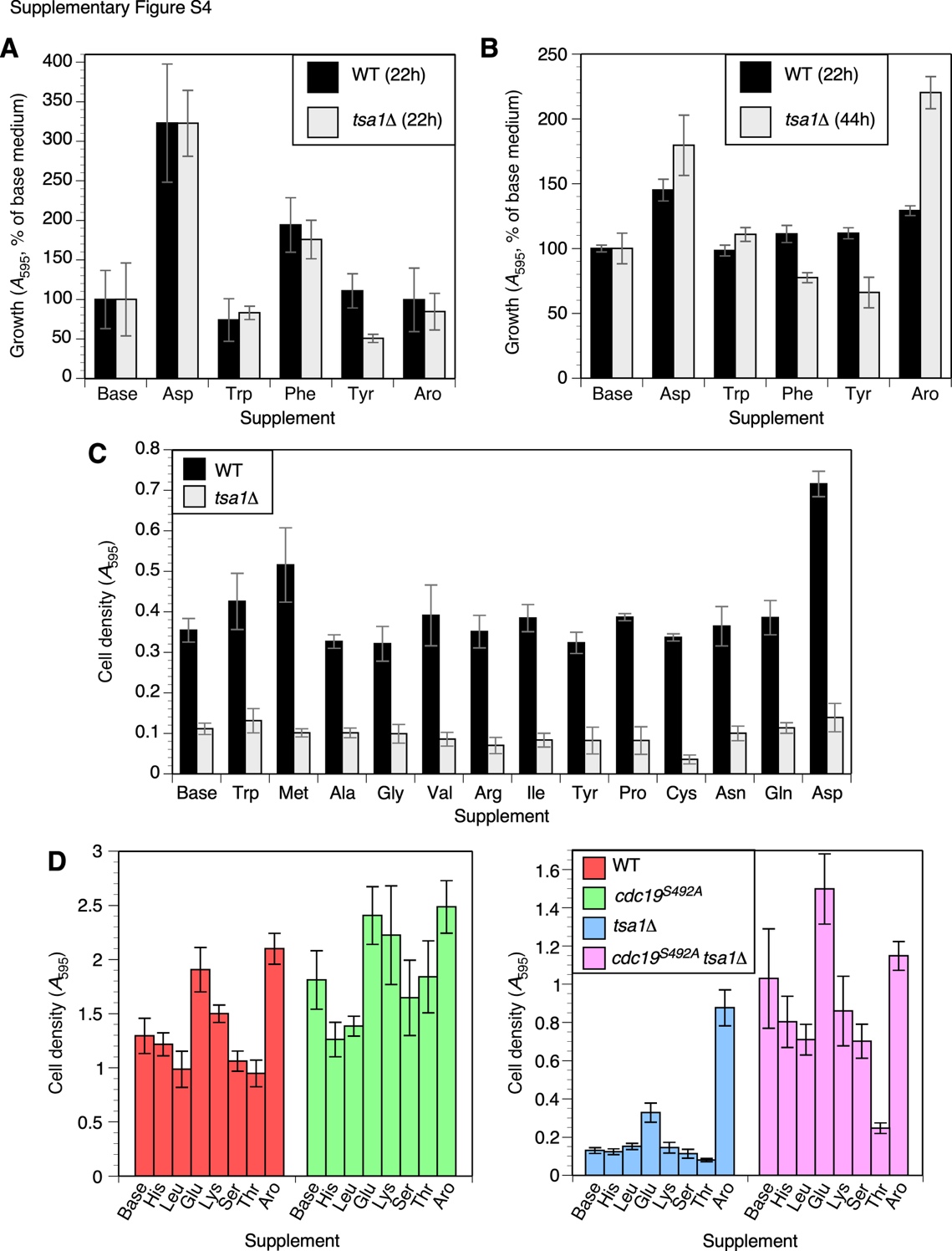
**

**Supplemental Fig. S4**: **Effect of amino acid supplementation on growth.**

A, B) Effect of aromatic amino acids and aspartate on growth in ZnR and ZnD medium. Cultures of wild-type BY4742 and *tsa1Δ* strains in LZM + 100 μM (panel A) and LZM + 1 μM (panel B) zinc were inoculated to a starting *A*_595_ of 0.01 and cultures grown for the indicated times. The slow growth of *tsa1Δ* strains in low zinc necessitated incubation for 44 h (panel B). Values are represented relative to the base medium, which contained lysine, leucine, uridine, and histidine to support strain auxotrophies, and was supplemented with 0.01% w/v of additional amino acids as indicated (Aro = 0.01% phenylalanine, tyrosine and tryptophan).

C) Growth of strains of the indicated genotypes (BY4742 and BY4742 *tsa1Δ*) in LZM + 1 μM zinc containing auxotrophic supplements (base, 0.01% w/v uracil, histidine, leucine, and lysine) or additionally supplemented with 0.01% w/v of the indicated amino acids. Cultures were inoculated to a starting *A*_595_ of 0.01 and grown for 2 days.

D) Growth of prototrophic strains of the indicated genotypes (CWM313, 314, 327 and 329 transformed with pHLK) in LZM + 1 μM zinc without supplements (base) or supplemented with 0.01% w/v of the indicated amino acids. *TSA1* wild-type strains were measured after 2 days (*left* panel), and *tsa1Δ* strains after four days (*right* panel) to more clearly reveal effects of the supplements. All values are the average of three experimental replicates and error bars indicate ± 1 SD.


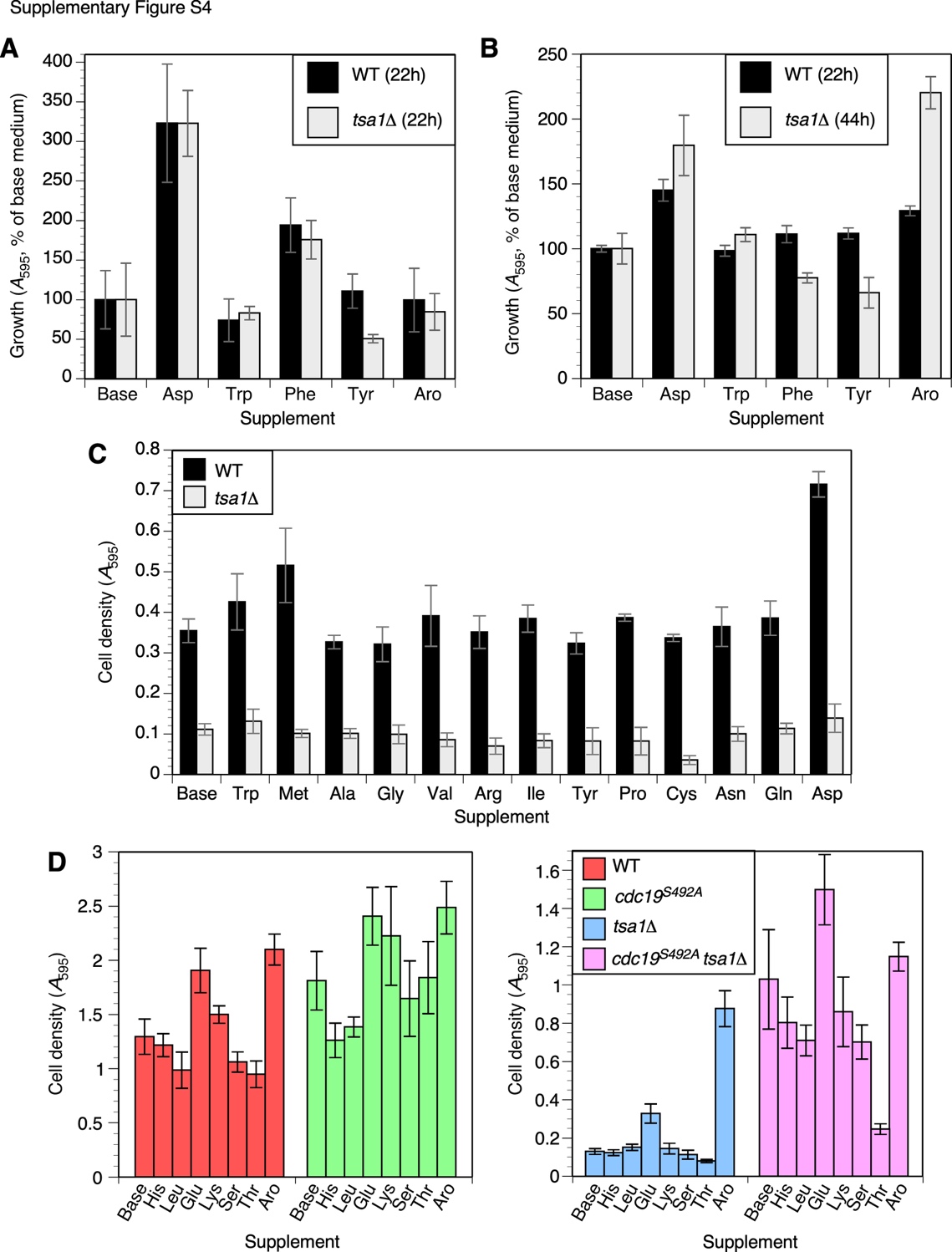


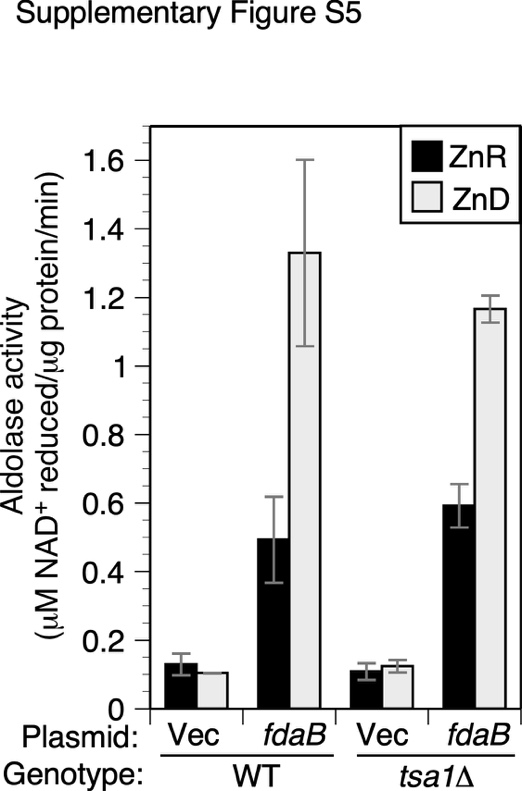


**Supplemental Figure S5: FdaB expression elevated zinc-independent aldolase activity in ZnD cells.**

Yeast strains (BY4742 and BY4742/*tsa1Δ*) were grown in in LZM + 100 μM (ZnR) or 1 μM (ZnD) zinc as described for **Fig. 6**, protein extracted, and aldolase assays performed without zinc addition. Values are the average of three experimental replicates and the error bars indicate ± 1 SD.


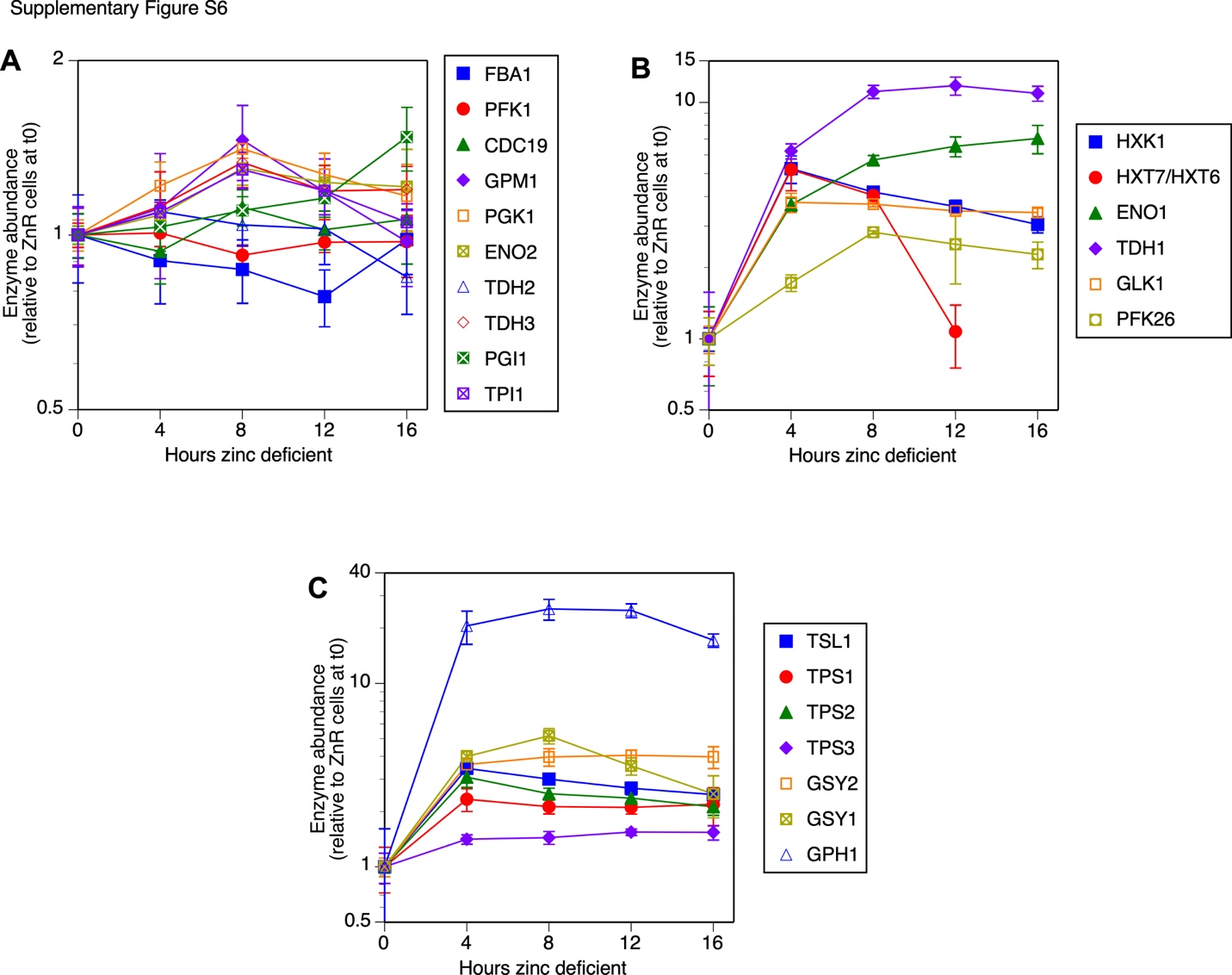


**Supplemental Figure S6: Changes in glycolytic and storage carbohydrate enzyme abundance upon induction of zinc deficiency.**

Wild-type yeast were grown in LZM + 100 μM zinc to log phase and transferred to zinc-deficient medium (LZM + 1 μM zinc), and samples taken at four hour intervals for LC-MS/MS proteomic analysis. Values shown are the average of LC-MS/MS peak intensity values (four experimental replicates), normalized to the values recorded for ZnR cells at time zero. Error bars represent ± 1 SD. A) Glycolytic enzymes showing < 2-fold change. B) Glycolytic enzymes showing >2-fold change. C) Storage carbohydrate (trehalose and glycogen) enzymes. Full dataset and details of the experiment were described previously (5).

**Table S1 Single nucleotide polymorphisms unique to the *stz3* gamete pool**

| **Chromosome** | **Position** | **Reference base** | **Variant allele** | **Frequency** | **Sequence Counts** | **Annotations** | **Amino Acid Change** |
| --- | --- | --- | --- | --- | --- | --- | --- |
| I | 73259 | T | G | 100 | 272 | CDS: CDC19 | Ser492Ala |
| II | 389430 | G | C | 100 | 9 | Replication origin: ARS213 | - |
| IV | 1525363 | C | G | 100 | 18 | Telomeric Region | - |
| V | 115962 | T | A | 100 | 12 | - | - |

**Table S2 Yeast strains used.**

| **Strain** | **Relevant genotype** | **Full genotype** | **Reference** |
| --- | --- | --- | --- |
| BY4741 | WT | *MAT*a *his3∆0 leu2∆0 met15Δ0 ura3∆0* | (66) |
| BY4742 | WT | *MAT*a *his3∆0 leu2∆0 lys2Δ0 ura3∆0* | (66) |
| BY4743 | WT | *MAT*a*/MAT*α *his3Δ1/his3Δ1 leu2Δ0/leu2Δ0 met15Δ0/MET15 lys2Δ0/LYS2 ura3Δ0/ura3Δ0* | (66) |
| CWY2 | WT | *MAT*α *his3Δ1 leu2Δ0 ura3Δ0* | (23) |
| CWY8 | *tsa1Δ* | *MAT*α *tsa1::KanMX4 his3Δ1 leu2Δ0 ura3Δ0* | (23) |
| BY4742/*tsa1Δ* | *tsa1Δ* | *MATa tsa1::KanMX4 his3∆0 leu2∆0 lys2Δ0 ura3∆0* | (67) |
| CWY10 | *tsa1Δ* *tsa2Δ* | *MAT*a *tsa1::KanMX4 tsa2::KanMX4 his3Δ1 leu2Δ0 ura3Δ0* | (23) |
| 23-1D | *tsa1Δ* *cdc19^S492A^* | *MAT*α *tsa1::KanMX4 cdc19^S492A^ his3Δ1 leu2Δ0 ura3Δ0 Tn-LEU2** | This work |
| 23-1D/22 | *tsa1Δ* *cdc19^S492A^* | *MAT*a *tsa1::KanMX4 cdc19^S492A^ his3Δ1 leu2Δ0 lys2Δ0 ura3Δ0* | This work |
| CWM83 | *trr1Δ* *tsa1Δ* | *MAT*α *tsa1::LEU2 trr1::KanMX4 his3Δ1 leu2Δ0 ura3Δ0* | This work |
| MDY2 | *tsa1Δ* *tsa2Δ* *cdc19^S492A^* | *MAT*a *tsa1::KanMX4 tsa2::HphMX4 cdc19^S492A^ his3Δ1 leu2Δ0 lys2Δ0 ura3Δ0* | This work |
| CWM307 | *cdc19Δ* | *MATa cdc19::KanMX4 his3∆0 leu2∆0 lys2Δ0 ura3∆0* | This work |
| CWM313 | *CDC19* | *MATa cdc19::KanMX4 his3∆0 leu2∆0 lys2Δ0 ura3∆0 pCDC19* | This work |
| CWM314 | *cdc19^S492A^* | *MATa cdc19::KanMX4 his3∆0 leu2∆0 lys2Δ0 ura3∆0* p*cdc19^S492A^* | This work |
| CWM327 | *tsa1Δ* | *MATa cdc19::KanMX4 tsa1::LEU2 his3∆0 leu2∆0 lys2Δ0 ura3∆0 pCDC19* | This work |
| CWM329 | *tsa1Δ* *cdc19^S492A^* | *MATa cdc19::KanMX4 tsa1::LEU2 his3∆0 leu2∆0 lys2Δ0 ura3∆0* p*cdc19^S492A^* | This work |
| CWM331 | *cdc19Δ TEFpr-CDC19* | *MATa cdc19::KanMX4 his3∆0 leu2∆0 lys2Δ0 ura3∆0 pTEFpr-CDC19* | This work |
| CWM333 | *cdc19Δ TEFpr-PYK2* | *MATa cdc19::KanMX4 his3∆0 leu2∆0 lys2Δ0 ura3∆0 pTEFpr-PYK2* | This work |
| CWM337 | *cdc19Δ tsa1Δ* *TEFpr-CDC19* | *MATa cdc19::KanMX4 tsa1::LEU2 his3∆0 leu2∆0 lys2Δ0 ura3∆0 pTEFpr-CDC19* | This work |
| CWM339 | *cdc19Δ tsa1Δ* *TEFpr-PYK2* | *MATa cdc19::KanMX4 tsa1::LEU2 his3∆0 leu2∆0 lys2Δ0 ura3∆0 pTEFpr-PYK2* | This work |
| TH4015 | *tet0_7_-CDC19* | *MATa kanR-tet0_7_-TATA-CDC19 URA3::CMV-tTA his3-1 leu2*Δ*0 met15*Δ*0* | (40) |
| CWM343 | *tet0_7_-CDC19 tsa1Δ* | *MATa tsa1::LEU2 kanR-tet0_7_-TATA-CDC19 URA3::CMV-tTA his3-1 leu2*Δ*0 met15*Δ*0* | This work |
| CWM370 | *fba1Δ PGK1pr-FdaB* | *MATa fba1::KanMX4 his3∆0 leu2∆0 lys2Δ0 ura3∆0 pPGK1pr-FdaB* | This work |

*A *LEU2*-marked transposon insertion is present in 23-1D. Its location could not be definitively assigned by sequence as it is located in a repetitive region adjacent to either *PAU1* (Chr X) or *PAU14* (ChrIX).

**Table S3. Plasmids used.**

| **Plasmid** | **Description** | **Reference** |
| --- | --- | --- |
| pFL38 | *URA3*/*CEN* low copy shuttle vector. | (68) |
| pFL44-S | *URA3*/2 micron high copy shuttle vector. | (68) |
| pJAW79 | Yap1 *lacZ* reporter gene, Yap1-regulated element from *YCF1* promoter inserted in minimal *CYC1* promoter-*lacZ* fusion. | (73) |
| pCDC19 | *CDC19* genomic clone in pFL38. | This work |
| pcdc19^S492A^ | pCDC19 with S492A mutation. | This work |
| pRS313-HA-CDC19 | HA-tagged *CDC19* genomic clone in pRS313. | (43) |
| p413TEF-CDC19 | TEF promoter-*CDC19* fusion. | (42) |
| p413TEF-PYK2 | TEF promoter-*PYK2* fusion. | (42) |
| pHLK | Plasmid with *HIS3*, *LEU2* and *LYS2.* | (74) |
| pFdaB | *PGK1* promoter-FdaB-*CDC19* terminator in pFL44-S. | This work |
